# Explainable Deep Reinforcement Learning for Anomaly Detection in IoT-Enabled Metaverse Healthcare: Toward Trustworthy Cyber Threat Intelligence

**DOI:** 10.34133/research.1245

**Published:** 2026-04-23

**Authors:** Jing Yang, Xu Xu, Muhammad Attique Khan, Ghassen Ben Brahim, Jamel Baili, Lip Yee Por, Congsheng Li

**Affiliations:** ^1^ Center of Research for Cyber Security and Network (CSNET), Faculty of Computer Science and Information Technology, Universiti Malaya, Kuala Lumpur 50603, Malaysia.; ^2^ School of Computer Science and Engineering, Northeastern University, Shenyang 110004, China.; ^3^ Center of AI, Prince Mohammad Bin Fahd University, Al-Khobar, Saudi Arabia.; ^4^ Department of Computer Engineering, College of Computer Science, King Khalid University, Abha 61413, Saudi Arabia.; ^5^ China Telecommunication Technology Laboratory, China Academy of Information and Communications Technology, Beijing 100191, China.

## Abstract

The dynamic metaverse paradigm integrates emerging technologies and offers transformative opportunities to enhance consumer healthcare applications through immersive, connected experiences. However, this paradigm faces substantial cybersecurity challenges, such as distributed denial-of-service attacks, probing, and port scanning. This undermines the trustworthiness and resilience of healthcare analytics frameworks. To address these threats, intrusion detection systems that support proactive anomaly detection are essential for securing metaverse-based healthcare applications. Conventional anomaly detection techniques face challenges such as low interpretability, suboptimal feature selection, class imbalance, and inefficient hyperparameter tuning. These challenges limit their reliability in practical cyber threat intelligence settings. To solve these challenges, this paper presents an anomaly-detection framework for Internet of Things-enabled metaverse healthcare environments. The proposed framework leverages an off-policy proximal policy optimization (PPO) algorithm that incorporates SHapley Additive exPlanations-based feature selection and class-specific reward adjustments to address imbalance. The reinforcement learning-based off-policy PPO enables adaptive, sample-efficient learning by leveraging prior experience during policy updates. The hyperparameters of the model are optimized using the Bayesian Optimization Hyperband algorithm to accelerate training and enhance performance. This optimization technique combines Bayesian search with the Hyperband method to improve efficiency and convergence during model tuning. The performance of our model is evaluated on NSL-KDD, MAWI, and CICIoT2023 datasets. The results depict that the model outperformed its contemporaries with state-of-the-art results where accuracy, F-measure, G-means, and area under the curve reached 88.005%, 87.271%, 87.986%, and 0.870; 92.184%, 88.992%, 89.738%, and 0.873; and 89.368%, 88.312%, 89.039%, and 0.836, respectively. The results confirm the effectiveness of the framework in cyber threat scenarios. They also show their potential for explainable, trustworthy intelligence in metaverse healthcare.

## Introduction

### Background

Immersive technologies, such as the metaverse, are advancing rapidly, attracting attention from academia and industry. They promise to improve healthcare and support trustworthy digital services in clinical and wellness contexts [1]. The metaverse systems combine augmented reality, virtual reality (VR), and social platforms with the Internet of Things (IoT), enabling highly interactive and interconnected consumer environments that demand secure, low-latency communication. This convergence supports timely diagnostics and physical rehabilitation by enabling real-time data analytics and fostering resilient, responsive healthcare systems. Such integration ensures ultra-reliable, low-latency communication among IoT devices, boosting productivity and automation in digital healthcare [2]. Consequently, IoT-enabled metaverse applications can deliver more efficient health analytics, though they also introduce new vectors for cyber threats and trust concerns [3]. However, these systems handle sensitive personal health data, making them vulnerable to cyber threats such as denial-of-service (DoS) attacks, probing, and scanning. Such threats can erode system trustworthiness and reduce consumer confidence [4]. Proactive defense mechanisms such as intrusion detection systems (IDS) are employed to monitor traffic and identify anomalies that signal potential threats [5]. Recent studies highlight the value of anomaly detection in reinforcing security and real-time resilience in virtualized and interconnected healthcare ecosystems [3,6].

In recent years, various deep learning (DL) methods for metaverse healthcare have been applied to anomaly detection [7–9]. DL models process data through multiple layers, allowing them to learn complex patterns directly from raw input. However, DL methods struggle with feature selection, class imbalance, and hyperparameter tuning, all of which can substantially reduce their performance. Accurate feature selection helps isolate relevant information from large datasets. Addressing class imbalance is important to ensure that rare but critical anomalies are not ignored. Efficient hyperparameter tuning further improves detection performance in diverse environments. This study proposes a DL-based anomaly detection model for metaverse healthcare, explicitly designed to overcome these challenges.

Selecting relevant features is essential for improving DL performance in anomaly detection tasks [10]. In many cases, datasets contain numerous variables that may obscure meaningful patterns if not properly managed [11]. Although DL models can learn features automatically, incorporating a feature selection process remains important to reduce overfitting and avoid capturing noise. This helps maintain generalization across different datasets. Feature selection can lower input dimensionality, improve model efficiency, and shorten training time [12]. It also enhances transparency, which is crucial in metaverse healthcare where interpretability and regulatory compliance are key. However, many existing feature selection methods struggle to scale or adapt in dynamic environments where data characteristics frequently change [13].

To address class imbalance, researchers have taken actions at both the data and algorithm levels. At the data level, techniques such as oversampling the minority class and undersampling the majority class are commonly used. For example, the synthetic minority over-sampling technique (SMOTE) creates new samples by interpolating nearby minority instances. As an undersampling example, the NearMiss method selects majority class samples based on their distance to the minority class [14,15]. However, data-level methods may lead to overfitting or to the discarding of valuable data. At the algorithm level, approaches such as cost-sensitive learning, ensemble methods, and decision threshold adjustment are employed. Cost-sensitive learning increases penalties for misclassifying minority instances. Ensemble methods combine outputs from multiple submodels to improve overall performance. Adjusting the decision threshold can help balance predictions during testing. However, focusing too heavily on the minority class may reduce the ability of the model to correctly classify majority class instances, leading to overall performance degradation [16].

Deep reinforcement learning (DRL) addresses feature selection and class imbalance through a reward-based mechanism [17]. This framework emphasizes critical yet frequently ignored attributes by assigning higher rewards to meaningful characteristics. Such a strategy improves the capacity of the model to recognize nuanced and vital data patterns. Adding explainable artificial intelligence methods, such as SHapley Additive exPlanations (SHAP) scores, to the DRL reward function further sharpens the ability of the model to prioritize useful features. SHAP scores measure the impact of each input feature on the model output. This allows the system to highlight the most influential variables. This focused weighting improves both the precision of feature selection and the effectiveness of the overall model. To address class imbalance, DRL increases rewards for correct predictions on minority classes, improving their accuracy without sacrificing overall performance. This adaptability makes DRL more responsive to dynamic data and complex relationships compared to traditional models. However, DRL often struggles with utilizing off-policy data effectively [18]. Off-policy proximal policy optimization (PPO) uses trust-aware reinforcement learning to adaptively handle threats by learning from past data, crucial for proactive cyber defense [19]. By drawing from a replay buffer, off-policy PPO enhances sampling efficiency and promotes diverse, adaptive learning strategies [20].

Selecting optimal hyperparameters is essential to enhance DL model performance and prevent overfitting or underfitting [21]. Since no universal configuration exists, different models require tailored tuning. Common optimization methods include grid search [22], Bayesian optimization (BO) [23], evolutionary algorithms [24], and Hyperband (HB) [25]. Grid search performs exhaustive combinations within a predefined range but is computationally expensive. BO uses probabilistic models to guide search more efficiently, though it may struggle with poorly chosen priors and local optima [26]. Evolutionary algorithms can handle complex spaces but require substantial time and resources. HB improves efficiency by quickly identifying and retaining promising configurations [27]. Bayesian Optimization Hyperband (BOHB) integrates Bayesian optimization and Hyperband, combining predictive accuracy with efficient resource allocation. BOHB improves hyperparameter tuning by focusing on promising regions. It quickly discards poor configurations, which is especially useful for complex tasks like anomaly detection [28].

### Related works

Integrating the metaverse into healthcare marks a revolutionary change, ushering in a new era where digital and physical realities merge in medical practice [29]. The metaverse is becoming a key part of the healthcare ecosystem. It creates new opportunities for better patient care, medical training, and collaboration across distant regions. The metaverse has vast applications in medicine, ranging from remote surgeries and patient monitoring to advanced simulation-based medical training and treatment planning [30]. These applications demand robust connectivity and enhanced security to protect data integrity and patient privacy, especially as 6G-enabled IoT technologies rise.

#### Machine learning

Various machine learning (ML) methods, such as support vector machines (SVMs), decision trees (DTs), random forests (RFs), and k-nearest neighbors (KNNs), have been applied to IoT anomaly detection. Dash et al. [31] used principal component analysis (PCA) to simplify data and enhance distributed denial-of-service (DDoS) detection. However, the model cannot adapt to evolving traffic patterns. Senthilkumar et al. [32] introduced the improved least-squares SVM (ILS-SVM) with Pearson correlation coefficient (PCC), which may fail to capture the nonlinear dependencies common in IoT data. Altulaihan et al. [11] combined correlation-based feature selection (CFS) with genetic algorithms, yet traditional classifiers often limit performance in complex environments. Saiyed and Al-Anbagi [33] proposed a genetic algorithm and *t* test for DDoS attack detection, although its reliance on predefined traffic volumes may reduce generalizability. Geetha et al. [34] applied adaptive weighted kernel SVM-based circle search for greater flexibility, but the high computational cost remains a challenge. Mehta et al. [35] evaluated ML and DL models for attack detection. However, their approach does not address class imbalance or continuous data drift, which are crucial in dynamic IoT contexts.

Anomaly detection in IoT healthcare has prompted various strategies targeting the challenges of real-time and imbalanced data. Wu et al. [3] proposed data stream anomaly detection (DS-AD) for metaverse healthcare, leveraging 6G and locality-sensitive hashing isolation forest with sliding-window updates. While effective in handling data stream variability, the reliance on hash-based isolation forests may reduce sensitivity to subtle anomalies. Abououf et al. [36] developed a lightweight autoencoder-based method with artificial neural network classification and kernel SHAP for explainability. Though the model is efficient and interpretable, a shallow architecture may limit its ability to capture complex clinical patterns. Subha and Sathiaseelan [37] addressed data imbalance using the anomaly detection method and the oversampling (ADO) framework. It combines clustering of lower and upper boundary standardisation (CLUBS) with oversampling to improve classifier performance. However, synthetic sample generation may introduce noise or lead to overfitting, especially in highly dynamic IoT health data.

Recently, Zhu et al. [38] developed an anomaly detection model for metaverse healthcare using 6G networks. It combines data fusion, blockchain, and an isolation forest optimized using particle swarm optimization (PSO). While it ensures real-time detection and data integrity, integrating multiple advanced modules may limit scalability. Khan and Alkhathami [39] applied RF and deep neural networks with feature correlation for IoT healthcare. However, the study lacks details on deployment latency and real-world performance. Kadir et al. [40] emphasized the early detection of anomalies in patient vitals using IoT sensors, offering practical clinical value. However, the model may struggle with data drift and require retraining. Rahman et al. [41] proposed a framework using KNN, RF, and DT to secure IoT medical devices. However, reliance on conventional classifiers could hinder the detection of advanced threats. Subha and Sathiaseelan [42] introduced a 3-phase method involving noise reduction, data balancing, and hybrid detection, effectively addressing real-world challenges. However, its multistage structure may incur high computational costs. Nasayreh et al. [43] combined long short-term memory (LSTM), KNN, and PCA into a layered model that improves interpretability and efficiency. Nevertheless, its scalability for more complex, multisource environments remains limited.

#### Deep learning

DL models, especially convolutional neural networks (CNNs) and LSTM networks, exhibit strong adaptability in IoT anomaly detection by learning complex spatial and temporal patterns. Saiyed and Al-Anbagi [44] developed a CNN-LSTM ensemble with unit pruning to balance accuracy and efficiency. Although effective against various DDoS attacks, its responsiveness in highly dynamic networks remains uncertain. Nandanwar and Katarya [45] proposed a CNN-gated recurrent unit (CNN-GRU) model for botnet detection in industrial IoT, highlighting its robustness in critical systems. However, its effectiveness against zero-day attacks remains unclear. Alshehri et al. [46] introduced a one-dimensional-CNN-LSTM (1D-CNN-LSTM) with skip connections optimized for fog environments and specific to Mirai and Bashlite attacks. However, this focus may limit generalization. e Huma et al. [47] designed a compact attention-based CNN with spatial attention for efficient detection on constrained devices. However, scalability across diverse protocols was not explored. Nazir et al. [48] combined CNN-LSTM with PCA, quantization, and pruning to improve deployment efficiency. However, its impact on fine-grained detection performance requires further analysis.

In healthcare-focused IoT anomaly detection, several studies have used DL to address complex threats. Gupta et al. [49] explored the use of CNNs for security in metaverse-based healthcare, highlighting their ability to handle high-dimensional data. However, the paper lacks a detailed evaluation under real-time or adversarial conditions. Varshney et al. [50] proposed LSTM-based models for anomaly detection in critical care, emphasizing improvements in patient outcomes. However, without model interpretability or clinical deployment tests, real-world applicability remains limited. Rajaprakash et al. [51] introduced a recurrent neural network (RNN)-based framework that integrates encryption and authentication for comprehensive IoT healthcare protection. However, its scalability and latency under resource constraints warrant further exploration. Khan et al. [52] developed a multilayer perceptron (MLP)-based IDS for smart healthcare, trained on public datasets. While effective in standard settings, the adaptability of the model to novel threats remains unclear. Guan et al. [53] proposed a 2-stage anomaly detection system combining DT, CNN, and bidirectional LSTM, enhanced by SMOTE and PSO. The layered structure aids performance, but added complexity may affect real-time efficiency in industrial IoT. Sun and Zhao [54] designed a TinyML-based CNN IDS for 6G edge environments, applying quantization and pruning to meet real-time demands. Despite impressive resource efficiency, the ability of the model to generalize across diverse network protocols remains uncertain. Lastly, Aversano et al. [55] simulated IoT traffic using the IoT Flock tool with constrained application protocol and message queuing telemetry transport protocols. This synthetic dataset supports testing with various models, but the fidelity to real-world traffic patterns and attack behaviors requires critical validation.

Table 1 presents a comprehensive review of state-of-the-art ML and DL methods for anomaly detection in IoT environments. Prior studies using ML models (e.g., GBDT, SVM, and XGBoost) have been effective for structured data but require extensive manual feature engineering. DL-based methods (e.g., CNNs, LSTMs, and autoencoders) improve scalability and automate feature learning. However, they often overlook 4 major challenges: low interpretability, effective feature selection, handling class imbalance, and effective hyperparameter tuning. Several DL-based anomaly detection studies have attempted to address challenges such as feature selection and class imbalance. For instance, Guan et al. [53] applied SMOTE to improve classification performance under imbalanced data. Nazir et al. [48] employed PCA and pruning to reduce input dimensionality and enhance feature learning. Despite their utility, these approaches suffer from marked limitations. SMOTE can introduce noisy or unrealistic samples, thereby distorting minority class boundaries. PCA is a linear transformation that may discard nonlinear but relevant features, limiting the model’s expressiveness. Moreover, existing models rarely integrate all 4 components within a unified framework.

**Table 1.** Comparison of ML and DL models for anomaly detection in IoT

Reference	Method	Contribution	Limitation
Dash et al. [31]	PCA, scaling, encoding	Demonstrates the impact of preprocessing on IoT security	Dependent on quality of network traffic data
Senthilkumar et al. [32]	PCC and ILS-SVM	Advances IoT security via refined classification	Relies on accurate data preprocessing
Altulaihan et al. [11]	ML + anomaly detection	Detects abnormal traffic in DoS scenarios	Depends on feature selection robustness
Saiyed and Al-Anbagi [33]	GA, *t* test, tree models	Improves DDoS detection via feature selection	Performance tied to data quality
Geetha et al. [34]	AWSVM-CS	Effectively identifies IoT threats	Depends on accurate feature selection
Mehta et al. [35]	ML + DL comparison	Improves IoT threat detection	Choice of algorithm varies by attack type
Wu et al. [3]	DS-AD, LSHiForest	Tackles dynamic metaverse healthcare data	Needs continuous data stream
Abououf et al. [36]	Explainable AI	Enhances patient monitoring in IoT	Requires high-quality consistent data
Subha and Sathiaseelan [37]	ADO with clustering of lower and upper boundary standardisation (CLUBS)	Better classification for imbalanced data	Relies on accurate anomaly detection
Zhu et al. [38]	6G + PSO-enhanced iForest	Secures metaverse healthcare data	Requires advanced network infra
Khan and Alkhathami [39]	ML for IoT healthcare	Boosts system security	Needs high-quality data streams
Kadir et al. [40]	IoT sensors + ML	Early detection of hospital anomalies	Depends on sensor data quality
Rahman et al. [41]	ML for intrusion detection	Secures medical IoT devices	Requires comprehensive input data
Subha and Sathiaseelan [42]	Noise reduction + data balancing	Reliable IoT health data analysis	Initial data quality critical
Nasayreh et al. [43]	KNN, LSTM, PCA	Improves cyber threat detection	Needs parameter tuning
Saiyed and Al-Anbagi [44]	CNN + LSTM + pruning	Efficient DDoS detection	Requires continual optimization
Nandanwar and Katarya [45]	CNN-GRU	Detects IoT botnet attacks	Needs large, high-quality dataset
Alshehri et al. [46]	1D-CNN + LSTM + skip	Botnet detection in constrained devices	Gating mechanism critical
e Huma et al. [47]	ACNN + spatial attention	Enhances detection efficiency	Requires advanced hardware
Nazir et al. [48]	CNN-LSTM + PCA/pruning	Improves IoT threat detection	Needs complex processing
Gupta et al. [49]	CNN for metaverse IoT	Handles high-dimensional data	Resource-intensive
Varshney et al. [50]	LSTM	Enhances healthcare data security	Needs scalability, interpretability
Rajaprakash et al. [51]	RNN + encryption	Reduces IoT false positives	Depends on real-time monitoring
Khan et al. [52]	MLP-based IDS	Protects smart healthcare systems	Needs large benchmark dataset
Guan et al. [53]	DT + CNN-BiLSTM + PSO	Improves IoT anomaly classification	Feature selection dependent
Sun et al. [54]	TinyCNN + pruning	Real-time detection in 6G	Needs strong edge devices
Aversano et al. [55]	IoT flock simulation tool	Enables privacy-aware traffic analysis	Uses synthetic data only

PCA, principal component analysis; PCC, Pearson correlation coefficient; SVM, support vector machine; ML, machine learning; DoS, denial of service; DDoS, distributed denial of service; AWSVM-CS, adaptive weighted kernel SVM-based circle search; DL, deep learning; IoT, Internet of Things; DS-AD, data stream anomaly detection; LSHiForest, locality-sensitive hashing isolation forest; AI, artificial intelligence; PSO, particle swarm optimization; iForest, isolation forest; KNN, k-nearest neighbor, LSTM, long short-term memory; CNN, convolutional neural network; BiLSTM, bidirectional LSTM; CNN-GRU, CNN-gated recurrent unit; ACNN, attention-based CNN; RNN, recurrent neural network; MLP, multilayer perceptron; IDS, intrusion detection system

To address these 4 gaps, we propose a unified anomaly detection model that combines SHAP (for interpretability), off-policy PPO (feature selection and class imbalance), and BOHB (for efficient hyperparameter tuning). This joint integration is novel in the context of anomaly detection and is tailored to the challenges of 6G-enabled IoT environments.

### Research contributions

This paper proposes a novel framework for anomaly detection in IoT-enabled metaverse health services. The off-policy PPO serves 2 roles. It is first combined with SHAP to enable interpretable feature selection. Second, it is enhanced with a custom reward function that improves performance on imbalanced data. BOHB is employed for efficient hyperparameter optimization. Prior studies have explored these components in isolation. However, no existing work has combined them into a unified architecture designed specifically for high-dimensional and imbalanced healthcare data in the metaverse. This combination fills a critical gap in current research by jointly enabling adaptability, explainability, and optimization, 3 pillars often treated separately in anomaly detection models. The main contributions of the research include the following:

1. This research adopts an off-policy PPO algorithm that uses both past and current policy data. This improves learning efficiency, stability, and generalization in metaverse healthcare.
2. SHAP values are integrated into the PPO reward function to guide feature selection. This boosts accuracy, lowers dimensionality, and improves interpretability in sensitive healthcare contexts.
3. To handle class imbalance, the model assigns higher rewards to minority-class predictions. This increases sensitivity to rare anomalies and reduces bias toward majority classes.
4. The BOHB algorithm is used for hyperparameter tuning. By combining BO with HB, it improves scalability and performance in dynamic 6G-IoT settings.

The paper is organized as follows: Results and Discussion examines the methodology proposed in this paper, describes the techniques and algorithms developed to improve anomaly detection, and presents the results of the experimental studies to assess the effectiveness of the proposed method across various scenarios. Conclusion concludes the paper by summarizing our findings and offering suggestions for future research in the area. Finally, Materials and Methods describes the datasets used and the evaluation metrics adopted in this study.

## Results and Discussion

### Structure of the proposed anomaly detection model

**Fig. 1.**
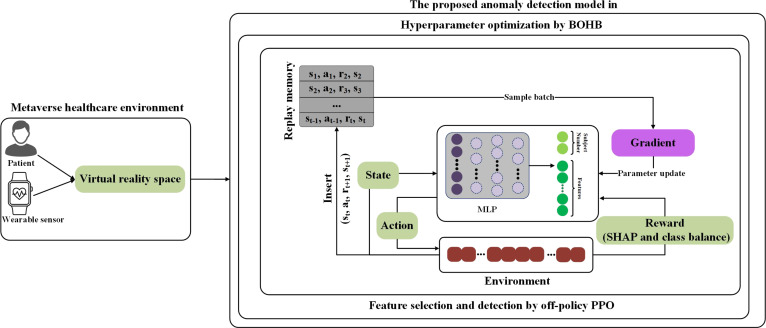
Structure of the proposed anomaly detection model in Internet of Things (IoT)-enabled metaverse healthcare. The framework employs an off-policy proximal policy optimization (PPO) for training. It utilizes a custom reward that incorporates SHapley Additive exPlanations (SHAP) values and prioritizes the minority class to enhance feature selection and address class imbalance. The Bayesian Optimization Hyperband (BOHB) algorithm is applied for efficient hyperparameter optimization.

Figure 1 depicts the overall architecture of the model for DDoS detection. In this model, an input consisting of *n* features is processed by the MLP network. This network is configured to produce *c* outputs, where *c* represents the categories (attack and nonattack). In this setup, an input layer that receives *n* feature channels feeds into an MLP network designed to produce c+n outputs. Here, c=2 corresponds to the classifications (anomaly and nonanomaly), and *n* indicates the number of input features.

#### Feature selection and detection

Consider the dataset *D* composed of pairs $\left(x,y\right)$, where *x* is a vector containing *n* input features $F=\left\{f_{1},\ldots ,f_{n}\right\}$, and *y* represents the target variable. In each iteration, a single pair is randomly selected from *D*, and decisions are made about which features to utilize. Within our RL framework:

• **State:** The state space $s_{t}$ represents the agent observation at timestep t. It includes the following: (a) the current data instance $x_{t}$, representing physiological or behavioral sensor readings from wearable or VR devices; (b) the score vector S produced by the MLP, encoding both feature relevance and predicted class labels; and (c) the active feature subset $F_{\text{active}}$, which evolves as the agent selects features sequentially. The state is designed to reflect characteristics of metaverse healthcare data. It captures patterns common in asynchronous, multimodal sensor streams, including delayed signals and short bursts of interaction. Overall, the state space dynamically encodes both static input characteristics and the history of agent decisions in environments with nonstationary and irregular data flows.
• **Action and reward:** The action set *A* in our model consists of 2 main types of actions: selecting a feature ($A_{f}$) or predicting an outcome ($A_{c}$). When a feature is selected from $A_{f}$, it expands the existing feature set *F*, thereby modifying the space for potential predictions. To encourage the selection of informative features, the agent receives a reward signal when a feature is selected. Specifically, the reward for feature selection is defined as $\lambda \times \text{SHAP}\left(f_{\text{best}}\right)$, where $\text{SHAP}\left(f_{\text{best}}\right)$ denotes the SHAP value of feature $f_{\text{best}}$, and λ serves as a balancing hyperparameter. Since SHAP values can be either positive or negative, this formulation naturally provides positive rewards for informative features and negative rewards for uninformative or harmful ones. The feature $f_{\text{best}}$ is identified by the MLP as having the highest score. Actions that lead to predictions move the model to a terminal state. The agent is rewarded or penalized with ±1 for correct or incorrect predictions for the majority class and ±γ for the minority class. The reward function r:S×A→R is structured to capture these interactions and incentives:

$$\begin{array}{c} r\left(\left(x,y,F\right),a\right)=\left\{\begin{array}{cc} \lambda \times \text{SHAP}\left(f_{\text{best}}\right) & \text{if}\;a\in A_{f},\;\\ +1 & \text{if}\;a\in A_{c},\;y\in D_{0},\;\hat{y}=y\\ -1 & \text{if}\;a\in A_{c},\;y\in D_{0},\;\hat{y}\neq y\\ +\gamma & \text{if}\;a\in A_{c},\;y\in D_{N},\;\hat{y}=y\\ -\gamma & \text{if}\;a\in A_{c},\;y\in D_{N},\;\hat{y}\neq y \end{array}\right. \end{array}$$ (1)

where $\hat{y}$ shows the predicted label for sample *x*. $D_{0}$ and $D_{N}$ denote the minority and majority classes, respectively. This reward system ensures an equitable learning approach that emphasizes accurate identification across different classes. It particularly focuses on the minority class to guarantee fair treatment across diverse groups.

**Fig. 2.**
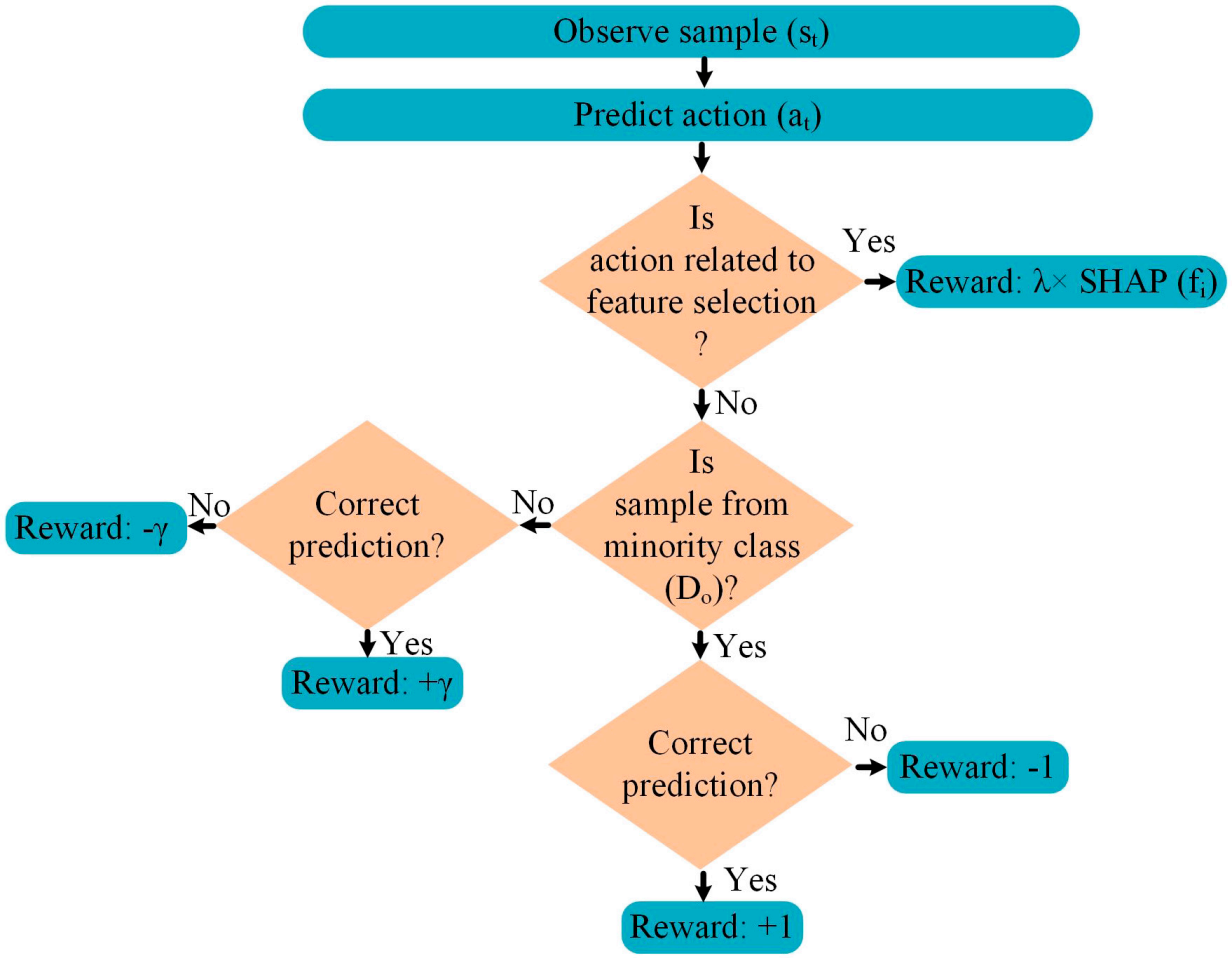
Flowchart of the reward mechanism used in the proposed reinforcement learning (RL) framework.

To better illustrate how the reward mechanism addresses feature selection and class imbalance, consider the flowchart in Fig. 2. The agent begins by observing a sample $s_{t}$ and predicting an action $a_{t}$. If the action corresponds to feature selection, the agent receives a reward defined as $\lambda \times \text{SHAP}\left(f_{i}\right)$, where $f_{i}$ is the selected feature. If the action instead corresponds to classification, the reward depends on the class label and prediction outcome. For the minority class $D_{0}$, the agent receives +1 for a correct prediction and −1 for an incorrect one. For the majority class $D_{N}$, the rewards are scaled to ±γ. This hierarchical reward structure avoids sign ambiguity, ensures consistent learning behavior, and promotes balanced, explainable classification.

The transition function of the model, designated as t:S×A→S∪T, operates as follows:

$$\begin{array}{c} t\left(\left(x,y,F\right),a\right)=\left\{\begin{array}{cc} T & \text{if}\;a\in A_{c}\\ \left(x,y,F\cup a\right) & \text{if}\;a\in A_{f} \end{array}\right. \end{array}$$ (2)

In this scenario, *T* signifies the terminal state. Whenever an action entails the addition of a new feature, that feature becomes part of the set *F*. The agent is restricted from reselecting previously chosen features, ensuring each $a\in A_{f}$ corresponds to a unique expansion of *F*. The episode concludes when the agent opts to make a prediction. After entering the terminal state *T*, a new sample $\left(x^{\prime },y^{\prime }\right)$ is drawn from the environment or replay buffer to initialize the next episode. The RL model operates in a discrete-time structure. The time variable *t* in $s_{t}$ and $s_{t+1}$ represents the moments when decisions are made to incorporate features. Each decision or timestep involves selecting a feature that can improve the accuracy of future predictions. This action directly influences the next state and reward. This continuous decision-making process is fundamental to RL and sets it apart from static supervised learning models, where feature sets are predetermined and not dynamically optimized.


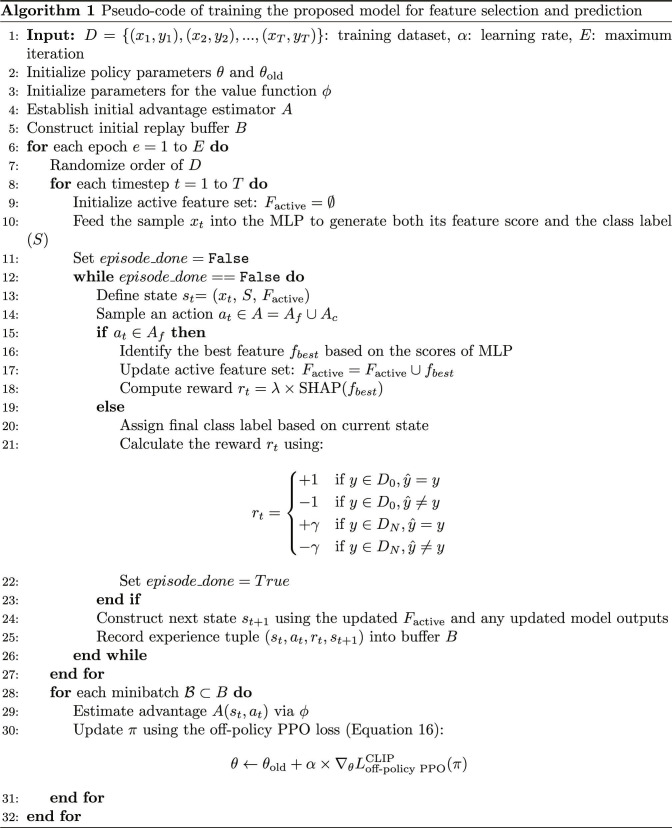


Algorithm 1 presents the training procedure of the proposed model, which jointly performs feature selection and classification. The process begins by initializing the policy network parameters (θ), value function parameters (ϕ), an advantage estimator (*A*), and a replay buffer (*B*). For each epoch, the dataset is shuffled to improve generalization. At each timestep, a data sample $x_{t}$ is passed through an MLP that outputs both a predicted class label (*S*) and feature importance scores. The model maintains an initially empty feature set. An action $a_{t}$ is then sampled from the combined space of feature-selection actions ($A_{f}$) and classification actions ($A_{c}$). If $a_{t}$ belongs to $A_{f}$, the model selects the most informative feature $f_{\text{best}}$ based on the MLP scores. The top-scoring feature is selected using the outputs of the MLP. However, the scoring function is updated through policy gradients using the off-policy PPO method. As a result, reinforcement signals guide and refine the feature selection process. This makes it an RL-based selection method instead of a fixed greedy approach. This feature is added to $F_{\text{active}}$, and the agent receives a reward proportional to its SHAP value. If $a_{t}$ belongs to $A_{c}$, the agent predicts $\hat{y}$ using the current state. The reward is calculated based on prediction accuracy and class imbalance, with a higher magnitude for minority-class outcomes. The episode then terminates. After each step, the transition tuple $\left(s_{t},a_{t},r_{t},s_{t+1}\right)$ is stored in buffer *B*, which accumulates experiences across the epoch. Once all training samples are processed, the model performs policy updates using minibatches sampled from *B*, applying the clipped loss function of the off-policy PPO method [Eq. (16)]. This iterative strategy allows the model to gradually refine its feature selection policy and classification performance in a unified learning loop. The buffer enables off-policy learning by decoupling data collection from policy updates.

### Training

To enhance policy training, our model adopts an off-policy PPO approach that combines the stability of trust region policy optimization (TRPO) and the efficiency of PPO. This section reviews the foundations of TRPO and PPO before detailing the proposed off-policy PPO strategy for effective policy refinement.

**Trust region policy optimization:** In conventional RL, the objective is to formulate a policy, π, aimed at maximizing the total discounted reward from the initial state. This is as outlined in the following [56]:

$$\begin{array}{c} \eta \left(\pi \right)=E_{s_{0},a_{0},\ldots }\left[R_{0}\right]=E_{s_{0},a_{0},\ldots }\left[\sum_{t=0}^{\infty }\gamma^{t}r(s_{t},a_{t})\right] \end{array}$$ (3)

Initially, $s_{0}$ is sampled from an initial state distribution $\rho_{0}$. At any given time *t*, the action $a_{t}$ and the state $s_{t}$ are determined by the policy $\pi \left(a_{t}|s_{t}\right)$ and the probabilities of transitioning to the next state $\mathbb{P}\left(s_{t+1}|s_{t},a_{t}\right)$. The discount factor γ is used to prioritize immediate rewards over distant ones. The aim is to enhance the effectiveness of the policy [$\eta \left(\pi \right)$].

To achieve this, the TRPO method utilizes data gathered while following the policy. It aims to increase policy performance by optimizing a surrogate objective within a bounded region for the Kullback–Leibler (KL) divergence [57].

$$\begin{array}{c} \max_{\pi }E_{s∼\rho_{\pi_{\mathrm{old}}},\;a\in \pi_{\mathrm{old}}}\left[\frac{\pi_{\theta }\left(a|s\right)}{\pi_{\theta_{\mathrm{old}}}\left(a|s\right)}A_{\pi_{\mathrm{old}}}(s,a)\right] \end{array}$$ (4)

Subject to:

$$E_{s∼\rho_{\pi_{\mathrm{old}}}}\left[D_{\mathrm{KL}}(\pi_{\mathrm{old}}\left(\cdot |s\right)\;∥\;\pi \left(\cdot |s\right))\right]\leq \delta$$ (5)

where $\pi_{\mathrm{old}}$ denotes the prior policy, and δ sets the limit for permissible divergence. The expression $D_{\mathrm{KL}}\left(\pi_{\mathrm{old}}\left(\cdot |s\right)\;∥\;\pi \left(\cdot |s\right)\right)$ quantifies the KL divergence, indicating the extent to which the new policy π deviates from the former policy $\pi_{\mathrm{old}}$ at any state *s*. The term $\rho_{\pi_{\mathrm{old}}}$ symbolizes the state probability distribution, originating from the initial state $s_{0}$ and adhering to the old policy $\pi_{\mathrm{old}}$, computed as $\rho_{\pi_{\mathrm{old}}}\left(s\right)=\sum_{t=0}^{\infty }\gamma^{t}\mathbb{P}\left(s_{t}=s|s_{0},\;\pi_{\mathrm{old}}\right)$.

Absent this divergence constraint, refining the surrogate objective function could lead to drastic alterations in the policy.

**Proximal policy optimization:** To inhibit extensive alterations in policy, PPO employs an adapted surrogate objective designed to be maximized. This revised objective, known as the clipped surrogate objective within PPO, is outlined as follows [58]:

$$\begin{array}{c} L_{\mathrm{PPO}}^{\text{CLIP}}\left(\theta \right)=E_{s∼\rho_{\pi_{\mathrm{old}}},\;a∼\pi_{\mathrm{old}}}\\ \left[\min(r_{\theta }\left(s,a\right))A_{\pi_{\mathrm{old}}}(s,a),\text{clip}(r_{\theta }(s,a),1-\varepsilon ,1+\varepsilon )A_{\pi_{\mathrm{old}}}\left(s,a\right)\right] \end{array}$$ (6)

The parameter ε, a modest positive number, is chosen to balance the preservation of policy stability and the promotion of exploration. The advantage function $A_{\pi_{\mathrm{old}}}\left(s,a\right)$ captures the benefit of taking *a* in state *s*. It compares the outcome of this action to the average result of all actions available in that state under policy $\pi_{\mathrm{old}}$. This function effectively assesses whether an action outperforms or underperforms the average expectations under the given policy.

Clipping is employed to restrain excessive updates to the policy during training, which helps achieve steadier advancement. This concept is elaborated on further below [58]:

$$\text{clip}\left(x,a,b\right)=\max\left(a,\min\left(b,x\right)\right)$$ (7)

In this scenario, *x* represents the variable under adjustment, constrained within the boundaries *a* and *b*, which delineate the minimum and maximum permissible values for *x*. Notably, the clipped surrogate objective is used to prevent overly large updates to the policy. It does this by penalizing changes that make the ratio $\frac{\pi \left(a|s\right)}{\pi_{\mathrm{old}}\left(a|s\right)}$ diverge too far from 1. However, a critical limitation of the PPO framework is its dependence on on-policy data, contributing to increased sample complexity. This reliance restricts the use of off-policy data and necessitates continuous interactions between the agent and its environment, complicating the learning process.

**Off-policy PPO:** This research introduces a refined PPO variant that enhances data efficiency by leveraging past policy experiences, unlike traditional on-policy PPO, which depends solely on current data. By incorporating diverse historical data, off-policy PPO accelerates learning and improves performance across varied scenarios, following a surrogate objective similar to off-policy TRPO.

$$\max_{\pi }E_{s∼\rho_{\mu },\;a\in \mu }\left[\frac{\pi \left(a|s\right)}{\mu \left(a|s\right)}A_{\pi_{\mathrm{old}}}\left(s,a\right)\right]$$ (8)

Subject to:

$${\bar{D}}_{\mathrm{KL}}^{\rho_{\mu }^{\text{sqrt}}}\left(\mu ,\pi_{\mathrm{old}}\right)\cdot {\bar{D}}_{\mathrm{KL}}^{\rho_{\mu }^{\text{sqrt}}}\left(\pi_{\mathrm{old}},\pi \right)+{\bar{D}}_{\mathrm{KL}}^{\rho_{\mu }}\left(\pi_{\mathrm{old}},\pi \right)\leq \delta$$ (9)

where

$$\rho_{\mu }\left(s\right)=\sum_{t=0}^{\infty }\gamma^{t}\mathbb{P}\left(s_{t}=s|s_{0},\mu \right)$$ (10)

$${\bar{D}}_{\mathrm{KL}}^{\rho_{\mu }}\left(\pi_{\mathrm{old}},\pi \right)=E_{s∼\rho_{\mu }}[D_{\mathrm{KL}}(\pi_{\mathrm{old}}\left(\cdot |s\right)∥\pi (\cdot s))]$$ (11)

$$\begin{array}{c} {\bar{D}}_{\mathrm{KL}}^{\rho_{\mu }^{\text{sqrt}}}\left(\mu ,\pi_{\mathrm{old}}\right)=E_{s∼\rho_{\mu }}[\sqrt{D_{\mathrm{KL}}\left(\mu \left(\cdot |s\right)∥\pi_{\mathrm{old}}\left(\cdot |s\right)\right)}] \end{array}$$ (12)

$${\bar{D}}_{\mathrm{KL}}^{\rho_{\mu }^{\text{sqrt}}}\left(\pi_{\mathrm{old}},\pi \right)=E_{s∼\rho_{\mu }}[\sqrt{D_{\mathrm{KL}}\left(\pi_{\mathrm{old}}\left(\cdot |s\right)∥\pi \left(\cdot |s\right)\right)}]$$ (13)

In this context, μ signifies the guiding policy. The off-policy objective in Eq. (8) aims to refine the surrogate function. However, without enforcing the constraint in Eq. (9), this refinement may lead to large and unstable policy updates. To mitigate this risk, we utilize the PPO clipping method, modifying the surrogate objective as follows:

$$L_{\mu }\left(\pi \right)=E_{s∼\rho_{\mu },\;a\in \mu }[\frac{\pi \left(a|s\right)}{\mu \left(a|s\right)}A_{\pi_{\mathrm{old}}}\left(s,a\right)]$$ (14)

Using $L_{\mu }\left(\pi \right)$ from Eq. (14), we define the clipped surrogate objective for off-policy data as follows:

$$\begin{array}{c} \begin{array}{c} \begin{array}{c} {\bar{L}}_{\mu }\left(\pi \right)=E_{s∼\rho_{\mu },\;a\in \mu }\\ \left[\min\left(\frac{\pi \left(a|s\right)}{\mu \left(a|s\right)}A_{\pi_{\mathrm{old}}}\left(s,a\right),\;\text{clip}\left(\frac{\pi \left(a|s\right)}{\mu \left(a|s\right)},\;1-\epsilon ,\;1+\epsilon \right)A_{\pi_{\mathrm{old}}}(s,a)\right)\right] \end{array} \end{array} \end{array}$$ (15)

The proportion $\frac{\pi \left(a|s\right)}{\mu \left(a|s\right)}$ often exceeds the interval 1−ϵ to 1+ϵ. As a result, the policy $\pi \left(a|s\right)$ tends to remain consistent when optimizing the clipped surrogate objective. To counteract this stagnation, the boundaries of the clipped objective, ranging from $\left(1-\epsilon \right)$ to $\left(1+\epsilon \right)$, are adjusted in Eq. (16) by incorporating a correction factor $\frac{\pi_{\theta_{\mathrm{old}}}\left(a|s\right)}{\mu \left(a|s\right)}$:

$$\begin{array}{c} \begin{array}{c} \begin{array}{c} L_{\mathrm{off}-\text{PolicyPPO}}^{\text{CLIP}}\left(\pi \right)=E_{s∼\rho_{\mu },\;a\in \mu }\\ \left[\min\left(\frac{\pi \left(a|s\right)}{\mu \left(a|s\right)}A_{\pi_{\mathrm{old}}}\left(s,a\right),\;\text{clip}\left(\frac{\pi \left(a|s\right)}{\mu \left(a|s\right)},\;\frac{\pi_{\theta_{\mathrm{old}}}\left(a|s\right)}{\mu \left(a|s\right)}\left(1-\epsilon \right),\;\frac{\pi_{\theta_{\mathrm{old}}}\left(a|s\right)}{\mu \left(a|s\right)}\left(1+\epsilon \right)\right)A_{\pi_{\mathrm{old}}}(s,a)\right)\right] \end{array} \end{array} \end{array}$$ (16)

**Fig. 3.**
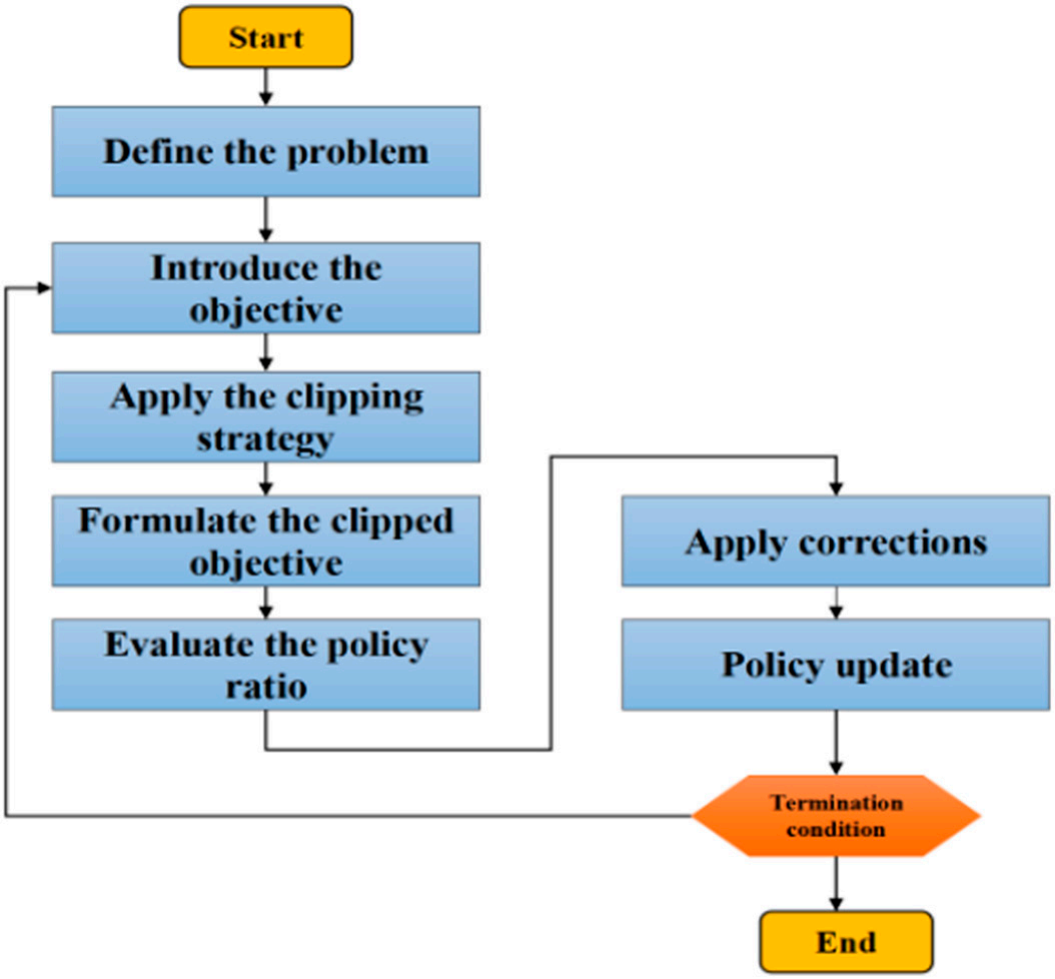
Seven-stage optimization framework for the anomaly detection model utilizing off-policy PPO.

Figure 3 delineates the procedure for optimizing the anomaly detection model utilizing off-policy PPO. The optimization starts by initializing the MLP network with prior data and previously learned policies. This initialization improves predictive accuracy, as shown in Eq. (8). A constrained surrogate objective is then applied to regulate policy updates, ensuring incremental changes for model stability [Eq. (14)]. To further control policy shifts, a clipping mechanism maintains update ratios within a safe range of 1−ϵ to 1+ϵ [Eq. (15)]. This clipped objective is adapted to incorporate off-policy data, allowing the model to leverage historical experiences without overfitting. The procedure verifies that updated policy ratios stay within the defined bounds. When deviations occur, a correction mechanism [Eq. (16)] adjusts them to prevent large shifts caused by outdated information. Together, these steps enable stable and efficient policy refinement using off-policy PPO. The concluding step updates the policy via the MLP network based on the optimized clipped objective. This process is repeated until the model achieves stable performance and improved accuracy.

### Hyperparameter optimization

Hyperparameter tuning plays a pivotal role in enhancing the efficacy of ML models. Imagine viewing an ML model as a function g:χ→ℝ, where χ encompasses all conceivable hyperparameters, with each potential configuration denoted by x∈χ. The goal of hyperparameter tuning is to identify the optimal set of hyperparameters, $x^{*}$, that optimizes the function $g\left(x\right)$.

However, for many ML models, directly determining $g\left(x\right)$ is not feasible due to the inherent variability and complexities involved. Typically, it is presumed that the function is accessible only through evaluations that are subject to noise [59,60]:

$$L_{\mu }\left(\pi_{i+1}\right)=y\left(x\right)=g\left(x\right)+\epsilon$$ (17)

In this context, ϵ signifies a noise factor adhering to a Gaussian distribution with a mean of zero and a variance denoted by $\sigma_{\text{noise}}^{2}$.

#### Bayesian optimization

BO [61] serves as a sophisticated approach for refining opaque black-box functions, which are generally costly and difficult to evaluate. This method proves especially beneficial for functions without clear analytical expressions, demanding extensive computational efforts for their evaluation. A key use of BO lies in the hyperparameter tuning of intricate ML models.

BO constructs a probabilistic model that estimates the performance of different inputs, thereby facilitating the identification of the most advantageous points for evaluation [62]. BO operates in cycles, creating a probabilistic model designated as $p\left(g|D\right)$ to predict the behavior of the target function *g* using Gaussian processes. This model leverages a dataset denoted as $D=\left\{\left(x_{0},y_{0}\right),\left(x_{1},y_{1}\right),\ldots ,\left(x_{i-1},y_{i-1}\right)\right\}$.

A crucial feature of this model is the acquisition function α:X→ℝ. It balances exploration of new regions and exploitation of promising areas using the latest updates from $p\left(g|D\right)$.

The iterative process includes the following steps:

• Identifying the optimal point $x_{\text{select}}$ where the acquisition function reaches its maximum, defined as:

$$x_{\text{select}}=\arg\max_{x\in X}\alpha \left(x\right)$$ (18)

• Determining the accurate value of the objective function at the chosen point is depicted as:

$$y_{\text{select}}=g\left(x_{\text{select}}\right)+\epsilon$$ (19)

• Incorporating the new observation into the current dataset *D*, thus updating the model with fresh data:

$$D=D\cup \left(x_{\text{select}},y_{\text{select}}\right)$$ (20)

• Each iteration builds on the previous, enhancing the precision of the model and its capacity to pinpoint the most advantageous observation, referred to as $x_{\text{best}}$. The overarching aim is to converge upon:

$$x_{\text{best}}=\arg\min_{x\in D}g\left(x\right)$$ (21)

In this context, BO iteratively models, evaluates, and refines to identify the optimal point for minimizing the objective function. This makes it a powerful tool for solving complex optimization problems with high computational efficiency.

#### Hyperband

HB [63] maximizes resource efficiency in hyperparameter optimization by using early termination to quickly eliminate poor configurations. This enables the faster identification and ranking of optimal settings across diverse scenarios.

#### BOHB hyperparameter optimization

BOHB refines hyperparameter optimization by combining the predictive power of BO (via Tree Parzen Estimator) with the efficient resource allocation of HB. This enables faster and more effective identification of promising configurations compared to traditional search methods. It also balances exploration and exploitation to navigate complex hyperparameter spaces effectively.

### Overall algorithm

**Fig. 4.**
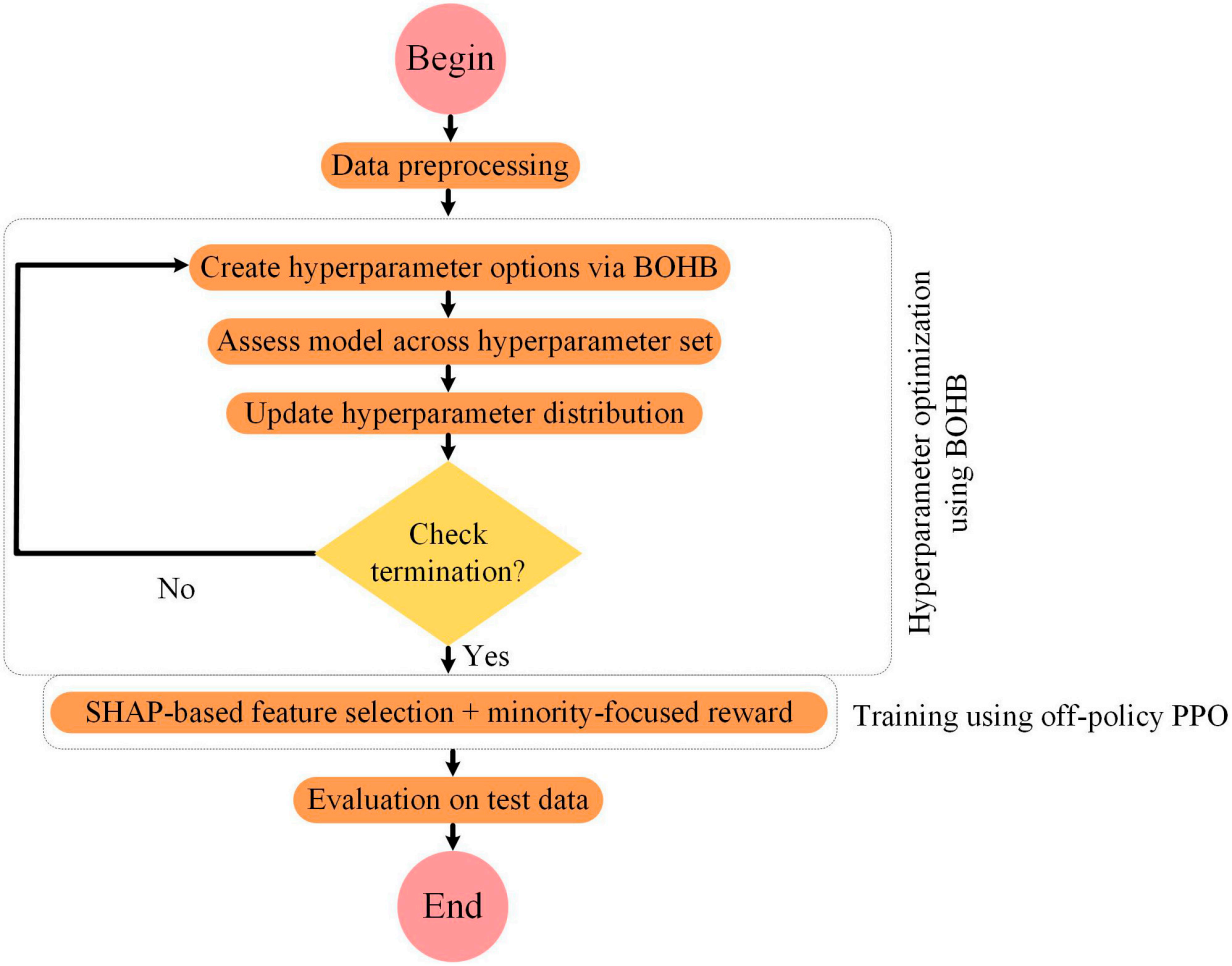
Overall flowchart of the proposed model.

Figure 4 presents the comprehensive pipeline of the proposed model. It includes 3 main stages: data preprocessing, hyperparameter optimization using BOHB, and reinforcement learning-based training via off-policy PPO. The pipeline begins with data acquisition. A multistage preprocessing routine is then applied to handle the diverse nature of metaverse healthcare data. First, missing value imputation is performed using either the mean or forward-fill strategy, depending on the signal type. This step addresses the common issue of data loss in sensor streams due to network latency or hardware failures in wearable or VR-based systems. Second, categorical features, such as user interaction types or virtual environment zones, are encoded using one-hot or ordinal encoding to make them compatible with neural models. Third, outlier detection is performed using interquartile range filtering. This removes anomalous values caused by sensor glitches or unrealistic avatar movements, which could otherwise distort training. Fourth, min–max scaling is applied to normalize continuous features to the range [0, 1]. This ensures consistent feature magnitudes, reduces bias from dominant variables, and accelerates convergence during optimization. Finally, temporal alignment is conducted when multimodal time-series inputs (e.g., physiological and behavioral signals) are involved. Synchronizing these streams ensures coherent state representation, which is critical for reinforcement learning-based models. These preprocessing steps are crucial for maintaining robustness and reliability in complex metaverse scenarios where input data can be asynchronous, incomplete, and noisy.

Following preprocessing, the model defines hyperparameter search spaces based on prior studies in anomaly detection within the metaverse healthcare context. These ranges are detailed in Table 2 and reflect empirically effective values for hyperparameters in similar domains. To optimize these configurations, BOHB is employed. BOHB runs for 20 iterations. In each iteration, it samples a set of hyperparameter configurations, trains each candidate for a budget between 1 and 256 epochs, and evaluates their performance. This multifidelity setup enables the low-cost early rejection of weak configurations, reserving extended training for only the most promising ones. In each round, new hyperparameter sets are generated and evaluated. The sampling distribution is updated based on performance, and this process continues until performance stabilizes. This budget-aware strategy directly addresses the reproducibility concern by clearly specifying the number of iterations, epoch bounds, and the allocation logic.

**Table 2.** Hyperparameters and their ranges for optimization in the model

Hyperparameter	Ranges	Best value
Batch size	[8, 512]	NSL-KDD (58), MAWI (52)
Epoch	[32, 1,024]	NSL-KDD (296), MAWI (258)
Learning rate	[0, 1]	NSL-KDD (0.03), MAWI (0.08)
Activation function	{Leaky ReLU, ReLU, Tanh, Linear, Sigmoid}	NSL-KDD (Leaky ReLU), MAWI (Leaky ReLU)
Dropout rate	[0, 1]	NSL-KDD (0.6), MAWI (0.5)
Layers in MLP	[1, 8]	NSL-KDD (5), MAWI (6)
Hidden size	[8, 1,024]	NSL-KDD (106), MAWI (114)
λ	[0, 1]	NSL-KDD (0.5), MAWI (0.3)
γ	[0, 1]	NSL-KDD (0.4), MAWI (0.5)

After BOHB completes, the best hyperparameter configuration is used for training. During this phase, the model performs SHAP-based feature selection and applies a reward strategy that focuses on identifying instances of the minority class. The trained model is finally evaluated on a held-out test set to assess generalization performance.

### Performance results of the proposed anomaly detection model

The experiments were conducted on a high-performance system with an Intel Core i7 central processing unit (CPU) and 32 gigabytes (GB) of random-access memory (RAM). An NVIDIA GTX 1080 Ti graphics processing unit (GPU) running on a 64-bit Windows platform was used to accelerate computations. Python and PyTorch were used for model development, with Stable Baselines for implementing off-policy PPO and HpBandSter for BOHB-based hyperparameter tuning. NSL-KDD and MAWI datasets were processed using Scikit-learn to ensure reproducibility and precision.

To ensure the reliability and robustness of the proposed model, we employ 5-fold stratified cross-validation. This method divides the dataset into 5 equal subsets, ensuring that each subset maintains the same proportion of normal and anomalous samples. In anomaly detection tasks with imbalanced data, stratification is essential. It ensures the model sees both normal and anomalous samples fairly during training and validation. This improves generalization and reduces the risk of bias toward majority class behaviors, which is vital in healthcare scenarios where false negatives could endanger patient safety. Final results are reported in the format of mean ± standard deviation, where the mean represents the average performance and the standard deviation reflects the consistency of results across folds.

In the evaluation phase, our model was extensively compared with 7 ML models, including ML-PCA [31], ILS-SVM [32], CFS-GA (genetic algorithm) [11], DS-AD [3], Explainable AI (EAI) [36], isolation forest (IF)-PSO [38], deep neural network (DNN) [39], and 6 DL models, including CNN-LSTM [44], CNN-GRU [47], deep convolutional neural network (DCNN) [49], LSTM-IoT [50], RNN-IoT [51], and Internet of Medical Things (IoMT) [52]. Additionally, we evaluated the impact of removing critical elements such as feature selection (FS), off-policy PPO, and hyperparameter optimization (HO) from our model to understand their contributions to overall performance. The outcomes of these comparisons, conducted on the NSL-KDD and MAWI datasets, are detailed in Table 3.

**Table 3.** Performance comparison of the proposed model against ML, DL, and ablated models using the NSL-KDD and MAWI datasets

Model	Accuracy	F-measure	G-means	AUC
**NSL-KDD**				
ML-PCA [31]	69.103 ± 0.003	69.343 ± 0.009	70.172 ± 0.035	0.683 ± 0.017
ILS-SVM [32]	70.467 ± 0.053	70.143 ± 0.034	70.998 ± 0.056	0.694 ± 0.032
CFS-GA [11]	71.021 ± 0.022	71.052 ± 0.043	71.954 ± 0.089	0.705 ± 0.048
DS-AD [3]	72.025 ± 0.093	72.638 ± 0.054	73.484 ± 0.018	0.719 ± 0.036
EAI [36]	73.247 ± 0.021	73.615 ± 0.060	74.468 ± 0.063	0.736 ± 0.027
IF-PSO [38]	74.216 ± 0.099	75.008 ± 0.060	75.885 ± 0.033	0.742 ± 0.072
DNN [39]	75.471 ± 0.081	76.363 ± 0.066	77.218 ± 0.060	0.765 ± 0.031
CNN-LSTM [44]	77.156 ± 0.100	78.563 ± 0.066	78.463 ± 0.074	0.773 ± 0.071
CNN-GRU [47]	77.742 ± 0.018	78.683 ± 0.076	79.593 ± 0.050	0.791 ± 0.023
DCNN [49]	79.096 ± 0.035	79.789 ± 0.042	80.648 ± 0.007	0.804 ± 0.088
LSTM-IoT [50]	80.561 ± 0.008	80.638 ± 0.001	81.480 ± 0.016	0.820 ± 0.071
RNN-IoT [51]	81.350 ± 0.009	81.471 ± 0.072	82.313 ± 0.069	0.829 ± 0.089
IoMT [52]	82.711 ± 0.039	82.881 ± 0.063	83.637 ± 0.013	0.846 ± 0.096
Proposed w/o FS	80.657 ± 0.090	81.048 ± 0.089	81.749 ± 0.042	0.822 ± 0.034
Proposed w/o PPO	84.363 ± 0.012	85.125 ± 0.006	84.790 ± 0.036	0.846 ± 0.038
Proposed w/o HO	86.745 ± 0.012	86.152 ± 0.006	86.909 ± 0.040	0.862 ± 0.057
**Proposed**	**88.005 ± 0.090**	**87.271 ± 0.033**	**87.986 ± 0.001**	**0.870 ± 0.011**
**MAWI**				
ML-PCA [31]	72.511 ± 0.056	72.202 ± 0.052	72.929 ± 0.032	0.702 ± 0.036
ILS-SVM [32]	73.690 ± 0.047	73.872 ± 0.021	74.517 ± 0.045	0.710 ± 0.034
CFS-GA [11]	74.589 ± 0.068	74.827 ± 0.036	75.512 ± 0.013	0.719 ± 0.030
DS-AD [3]	75.900 ± 0.047	75.949 ± 0.038	76.652 ± 0.080	0.729 ± 0.057
EAI [36]	77.346 ± 0.071	76.602 ± 0.098	77.285 ± 0.040	0.736 ± 0.050
IF-PSO [38]	79.154 ± 0.067	77.534 ± 0.023	78.282 ± 0.051	0.746 ± 0.013
DNN [39]	79.794 ± 0.068	77.998 ± 0.038	79.768 ± 0.084	0.762 ± 0.097
CNN-LSTM [44]	81.312 ± 0.053	80.762 ± 0.004	81.474 ± 0.023	0.775 ± 0.092
CNN-GRU [47]	82.674 ± 0.079	82.751 ± 0.023	82.751 ± 0.092	0.785 ± 0.066
DCNN [49]	84.037 ± 0.005	82.820 ± 0.027	83.456 ± 0.025	0.799 ± 0.051
LSTM-IoT [50]	85.460 ± 0.012	84.940 ± 0.006	84.906 ± 0.031	0.814 ± 0.066
RNN-IoT [51]	86.143 ± 0.091	83.360 ± 0.076	83.206 ± 0.094	0.822 ± 0.063
IoMT [52]	87.657 ± 0.064	86.560 ± 0.003	87.285 ± 0.010	0.839 ± 0.065
Proposed w/o FS	84.657 ± 0.064	82.560 ± 0.003	83.285 ± 0.010	0.804 ± 0.024
Proposed w/o PPO	88.261 ± 0.021	86.193 ± 0.001	86.909 ± 0.070	0.846 ± 0.088
Proposed w/o HO	90.665 ± 0.091	87.915 ± 0.021	88.604 ± 0.096	0.863 ± 0.038
**Proposed**	**92.184 ± 0.089**	**88.992 ± 0.089**	**89.738 ± 0.084**	**0.873 ± 0.010**

ML, machine learning; DL, deep learning; AUC, area under the curve; PCA, principal component analysis; CFS, correlation-based feature selection; DS-AD, data stream anomaly detection; PSO, particle swarm optimization; CNN, convolutional neural network; LSTM, long short-term memory; CNN-GRU, CNN-gated recurrent unit; IoT, Internet of Things; RNN, recurrent neural network; FS, feature selection; PPO, proximal policy optimization; HO, hyperparameter optimization

The proposed model demonstrated superior performance across all evaluation metrics, substantially outperforming state-of-the-art methods. It achieved an accuracy of 92.184%, surpassing IoMT (87.657%) and RNN-IoT (86.143%) by 4.53% and 6.04%, respectively. The model also achieved an F-measure of 88.992% and a G-mean of 89.738%. It indicates improved balance between precision and recall as well as heightened sensitivity to both majority and minority classes. Its area under the curve (AUC) reached 0.873, reflecting notable gains over IoMT (4.05%), RNN-IoT (5.17%), and EAI (13.69%). These improvements stem from the integration of off-policy PPO for minority class sensitivity and BOHB for efficient hyperparameter optimization.

The ablation study provided additional insights into the components of the model. When feature selection was removed (Proposed w/o FS), accuracy dropped by 7.52% to 84.657%, highlighting the critical role of SHAP-based selection in improving classification precision. Eliminating the off-policy PPO component (Proposed w/o PPO) led to a 4.14% reduction in accuracy, demonstrating its importance in addressing class imbalance and learning efficiency. Without BOHB hyperparameter optimization (Proposed w/o HO), performance declined by 1.43%, emphasizing BOHB’s contribution to model robustness and fine-tuning.

Overall, the proposed model consistently outperformed its ablated variants. It achieved F-measure gains of 7.42% and 4.64% over Proposed w/o FS and Proposed w/o PPO, respectively. The corresponding AUC improvements were 6.91% and 3.19%. These results affirm the complementary value of feature selection, off-policy RL, and efficient hyperparameter optimization in enhancing anomaly detection in complex IoT traffic environments.

Paired *t* tests were conducted to assess the statistical significance of performance gains achieved by the proposed model using the NSL-KDD and MAWI datasets. For NSL-KDD, the proposed model substantially outperformed IoMT, with *P* values below 0.001 for accuracy, F-measure, and G-means, and 95% confidence intervals showing accuracy improvements between 5.90% and 6.80%. On the MAWI dataset, *t* tests against both IoMT and RNN-IoT yielded *P* values below 0.01 across all metrics, with accuracy gains ranging from 4.50% to 6.20% and AUC improvements consistently exceeding 3.90%. These results confirm the statistical robustness of the proposed model, driven by the integration of off-policy PPO and BOHB optimization.

Table 4 compares the computational efficiency of the proposed model with 13 leading methods on the NSL-KDD and MAWI datasets, evaluating runtime and GPU usage. The reported memory usage reflects the combined consumption of system RAM and GPU VRAM, as measured by system-level profiling tools. This approach offers a holistic view of the actual memory footprint during end-to-end training and evaluation, better reflecting the practical resource needs in hybrid CPU–GPU environments. On NSL-KDD, it achieved 2,842 s and 22.9 GB—slightly above lightweight models like ML-PCA but more efficient than deep models like DCNN (2,963 s, 23.7 GB). For MAWI, the model ran in 2,903 s, using 23.8 GB, which reduced the runtime by 4.1% over IoMT and 5.9% over LSTM-IoT, with lower GPU usage. These results confirm the model’s efficiency and scalability, making it suitable for large-scale 6G-enabled IoT metaverse healthcare applications.

**Table 4.** Runtime and GPU memory comparison of the proposed model against ML and DL models using the NSL-KDD and MAWI datasets

Model	Runtime (s)	GPU memory (GB)
NSL-KDD		
ML-PCA [31]	1,982 ↓860	18.8 ↓4.1
ILS-SVM [32]	2,036 ↓806	20.1 ↓2.8
CFS-GA [11]	1,863 ↓979	18.4 ↓4.5
DS-AD [3]	2,145 ↓697	19.3 ↓3.6
EAI [36]	1,756 ↓1,086	20.4 ↓2.5
IF-PSO [38]	2,245 ↓597	19.6 ↓3.3
DNN [39]	1,904 ↓938	18.2 ↓4.7
CNN-LSTM [44]	2,465 ↓377	21.4 ↓1.5
CNN-GRU [47]	2,678 ↓164	22.6 ↓0.3
DCNN [49]	2,963 ↑121	23.7 ↑0.8
LSTM-IoT [50]	2,710 ↑132	25.9 ↑3.0
RNN-IoT [51]	2,971 ↑129	23.6 ↑0.7
IoMT [52]	3,045 ↑203	24.1 ↑1.2
**Proposed**	**2,842**	**22.9**
**MAWI**		
ML-PCA [31]	2,145 ↓758	19.4 ↓4.4
ILS-SVM [32]	2,163 ↓740	22.6 ↓1.2
CFS-GA [11]	1,942 ↓961	19.3 ↓4.5
DS-AD [3]	2,214 ↓689	21.4 ↓2.4
EAI [36]	2,004 ↓899	22.9 ↓0.9
IF-PSO [38]	2,347 ↑556	20.4 ↓3.4
DNN [39]	2,145 ↓758	19.3 ↓4.5
CNN-LSTM [44]	2,596 ↑307	22.1 ↓1.7
CNN-GRU [47]	2,842 ↑61	23.0 ↑0.8
DCNN [49]	3,048 ↑145	24.7 ↑0.9
LSTM-IoT [50]	2,863 ↑40	26.2 ↑2.4
RNN-IoT [51]	2,863 ↑40	25.1 ↑1.3
IoMT [52]	2,963 ↑60	26.3 ↑2.5
**Proposed**	**2,903**	**23.8**

GPU, graphics processing unit; ML, machine learning; DL, deep learning; PCA, principal component analysis; CFS, correlation-based feature selection; DS-AD, data stream anomaly detection; PSO, particle swarm optimization; CNN, convolutional neural network; LSTM, long short-term memory; CNN-GRU, CNN-gated recurrent unit; IoT, Internet of Things; RNN, recurrent neural network

**Fig. 5.**
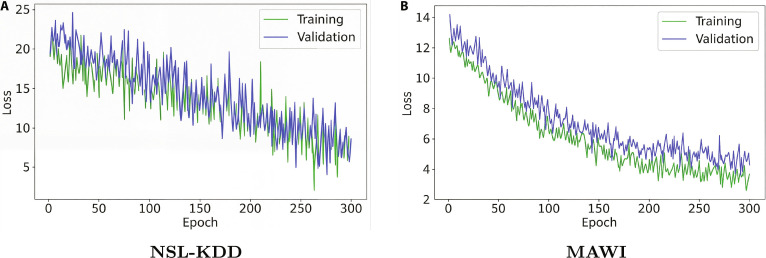
Training and validation loss curves for the (A) NSL-KDD and (B) MAWI datasets over 300 epochs.

Figure 5 presents the training and validation loss curves of the proposed model over 300 epochs for the NSL-KDD and MAWI datasets. The consistent decline and close alignment of both losses indicate effective convergence and strong generalization, with no signs of overfitting.

**Fig. 6.**
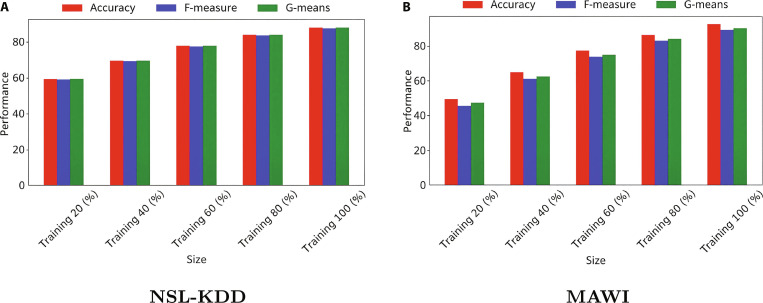
Demonstrating model scalability with increasing training data volume on the (A) NSL-KDD and (B) MAWI datasets.

Figure 6 shows that the proposed model scales effectively, with accuracy, F-measure, and G-means steadily improving as training data increases from 20% to 100% on NSL-KDD and MAWI. Performance gains are especially notable from 20% to 60%, and peak results are achieved with full data, demonstrating strong generalization and data efficiency. These trends confirm the model’s robustness and scalability for large-scale 6G-enabled IoT metaverse healthcare applications.

**Fig. 7.**
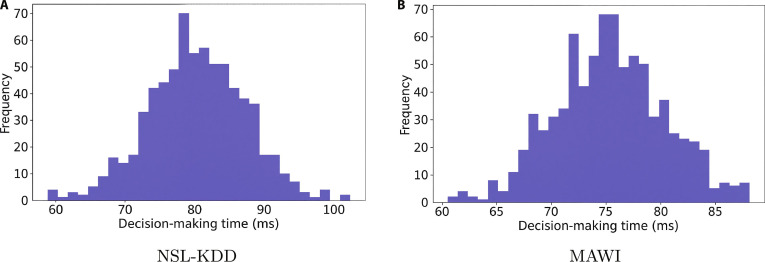
Decision-making time distributions for the proposed model on the (A) NSL-KDD and (B) MAWI datasets.

Figure 7 displays the decision-making time distributions of the proposed model on the NSL-KDD and MAWI datasets, showing consistently fast and stable performance. Times are tightly grouped—65 to 85 ms (mean ~75 ms) for NSL-KDD and 70 to 100 ms (mean ~80 ms) for MAWI. This indicates low variability and a reliable response. This consistency is critical for real-time IoT and healthcare applications. The model’s efficiency stems from optimized off-policy PPO and BOHB algorithms, enabling rapid, reliable detection in high-throughput metaverse environments.

#### Analysis of generalizability

We evaluated the proposed model on the CICIoT2023 dataset (Table 5). Traditional models like ML-PCA and ILS-SVM (67.39% and 68.06% accuracy) showed limited adaptability due to simplistic feature handling. DL models, including CNN-LSTM and RNN-IoT, performed better (up to 80.65%) by capturing temporal patterns, yet struggled with imbalanced data and optimization overhead.

**Table 5.** Performance comparison of the proposed model against ML and DL models using the CICIoT2023 dataset

Model	Accuracy	F-measure	G-means	AUC
ML-PCA [31]	67.393 ± 0.004	67.883 ± 0.071	68.695 ± 0.012	0.692 ± 0.045
ILS-SVM [32]	68.061 ± 0.063	69.311 ± 0.002	70.116 ± 0.009	0.701 ± 0.047
CFS-GA [11]	69.407 ± 0.005	70.500 ± 0.055	71.322 ± 0.003	0.707 ± 0.026
DS-AD [3]	71.080 ± 0.090	71.427 ± 0.053	72.247 ± 0.075	0.715 ± 0.044
EAI [36]	71.714 ± 0.064	72.506 ± 0.063	73.291 ± 0.031	0.731 ± 0.036
IF-PSO [38]	72.219 ± 0.024	73.498 ± 0.086	74.269 ± 0.020	0.757 ± 0.032
DNN [39]	72.870 ± 0.026	74.595 ± 0.046	75.362 ± 0.046	0.746 ± 0.036
CNN-LSTM [44]	77.335 ± 0.099	78.305 ± 0.052	78.979 ± 0.072	0.772 ± 0.050
CNN-GRU [47]	78.006 ± 0.081	79.020 ± 0.055	79.932 ± 0.081	0.766 ± 0.053
DCNN [49]	78.651 ± 0.073	79.931 ± 0.065	80.634 ± 0.073	0.784 ± 0.053
LSTM-IoT [50]	80.165 ± 0.016	81.034 ± 0.091	81.721 ± 0.061	0.799 ± 0.042
RNN-IoT [51]	80.655 ± 0.008	82.117 ± 0.082	82.517 ± 0.067	0.807 ± 0.044
IoMT [52]	82.271 ± 0.041	83.082 ± 0.083	83.849 ± 0.071	0.818 ± 0.002
**Proposed**	**89.368 ± 0.020**	**88.312 ± 0.006**	**89.039 ± 0.030**	**0.836 ± 0.011**

ML, machine learning; DL, deep learning; AUC, area under the curve; PCA, principal component analysis; CFS, correlation-based feature selection; DS-AD, data stream anomaly detection; PSO, particle swarm optimization; CNN, convolutional neural network; LSTM, long short-term memory; CNN-GRU, CNN-gated recurrent unit; IoT, Internet of Things; RNN, recurrent neural network

The proposed model achieved 89.37% accuracy, outperforming all baselines by a large margin. This superior performance stems from integrating off-policy PPO and BOHB, which enhance adaptability, feature selection, and hyperparameter tuning. Gains in F-measure and G-means confirm balanced, accurate detection across diverse IoT conditions.

These findings demonstrate the model’s robustness in handling complex, evolving IoT data, validating its real-world applicability and leadership in anomaly detection in IoT-enabled metaverse applications.

#### Analysis of off-policy PPO

**Fig. 8.**
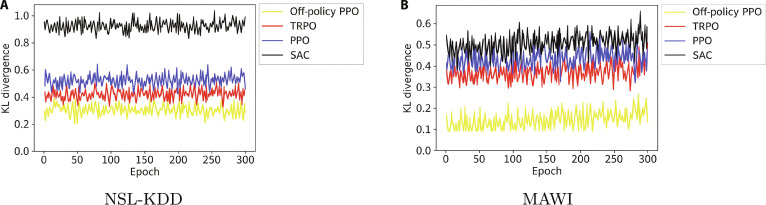
KL divergence comparison across reinforcement learning algorithms for the (A) NSL-KDD and (B) MAWI datasets in 300 epochs.

**Fig. 9.**
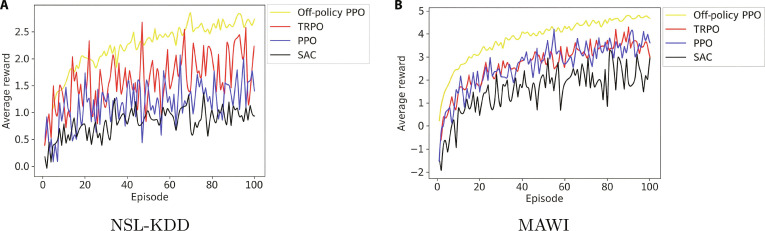
Average reward comparison across reinforcement learning algorithms for the (A) NSL-KDD and (B) MAWI datasets in 100 epochs.

To evaluate the stability of off-policy PPO, we compared it with PPO, TRPO, and soft actor–critic (SAC) using KL divergence and reward curves across the NSL-KDD and MAWI datasets (Figs. 8 and 9). SAC was included as a state-of-the-art off-policy baseline. Off-policy PPO consistently showed lower KL divergence, indicating smoother policy updates and greater training stability. This helps prevent disruptive shifts in policy behavior, which is crucial in critical real-time settings, such as 6G network security.

Reward analysis across 100 episodes further highlights off-policy PPO’s advantage, achieving higher and more stable returns than its counterparts. Its ability to leverage historical data, unlike on-policy methods, enhances learning efficiency, adaptability, and detection accuracy while reducing false positives.

These results confirm that off-policy PPO offers superior consistency, robustness, and scalability. This makes it highly suitable for real-world applications in dynamic environments such as IoT, healthcare, and metaverse cybersecurity systems.

**Fig. 10.**
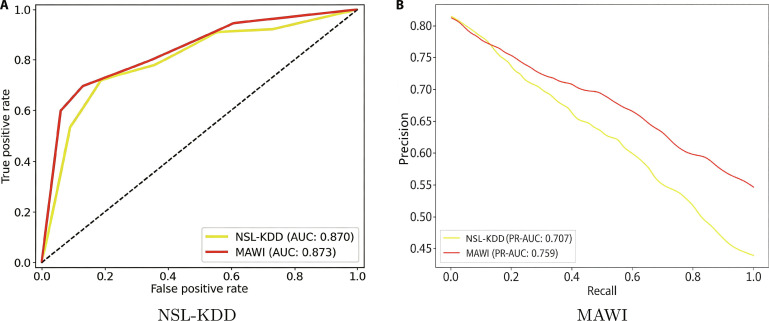
ROC and precision–recall (PR) curves of the proposed model on the (A) NSL-KDD and (B) MAWI.

Figure 10 illustrates the receiver operating characteristic (ROC) and precision–recall (PR) curves of the proposed model on the NSL-KDD and MAWI datasets, highlighting its effectiveness in imbalanced anomaly detection tasks. The ROC curves approach the top-left corner, with AUC scores of 0.870 (NSL-KDD) and 0.873 (MAWI), indicating high true positive rates and minimal false positives. PR-AUC scores of 0.707 and 0.759 further confirm the model’s strong precision–recall balance, especially important in datasets with scarce positive classes. The use of off-policy PPO enables the model to prioritize predictions for the minority class, thereby reducing false negatives while maintaining precision. This balanced performance across ROC and PR metrics underscores the model’s robustness and real-world applicability for timely and accurate anomaly detection in metaverse systems.

**Fig. 11.**
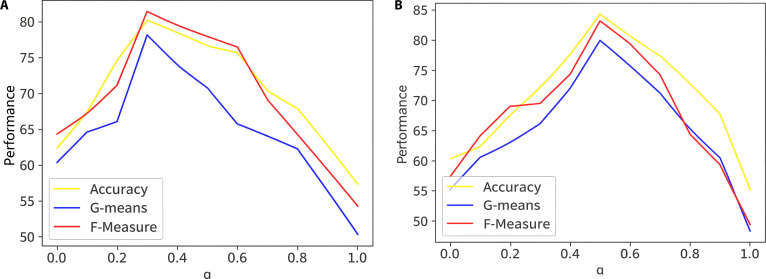
Impact of varying reward penalty parameter (μ) on the model performance for the (A) NSL-KDD and (B) MAWI datasets.

In the proposed model, rewards of +1 (minority) and μ (majority) are assigned for correct predictions, while misclassifications are penalized by −1 and −μ, respectively. The value of μ is tuned based on the class imbalance, with performance evaluated across μ values from 0 to 1 in 0.1 increments, as shown in Fig. 11 for NSL-KDD and MAWI datasets. For NSL-KDD, optimal performance occurs at μ=0.4, achieving a balanced sensitivity and specificity. For MAWI, μ=0.5 yields the best results, indicating that slightly stricter penalties on majority class errors improve precision. These settings substantially boost G-means and F-measure, reflecting better handling of class imbalance. Beyond these optimal points, performance drops, underscoring the need to finely tune μ based on data characteristics. Proper adjustment of this parameter ensures a strong trade-off between anomaly detection accuracy and minimizing false alarms. This is critical for reliable deployment in real-world metaverse IoT environments.

**Fig. 12.**
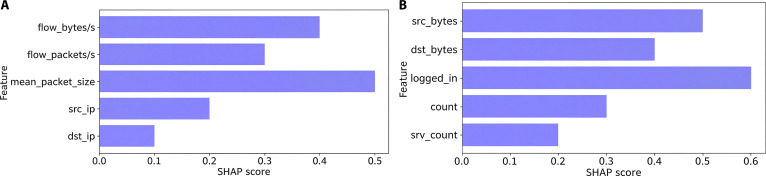
Comparative analysis of feature importance using the SHAP explanations on the (A) NSL-KDD and (B) MAWI datasets.

To enhance interpretability and validate feature selection, we employed SHAP, as shown in Fig. 12. SHAP values quantify each feature’s contribution to the model’s decisions, highlighting key predictors of anomalies in the NSL-KDD and MAWI datasets. The consistent importance of traffic-related features across both datasets confirms the robustness of our feature selection process. Moreover, SHAP enhances model transparency, a crucial quality for real-world deployment in critical 6G-enabled metaverse environments, where explainability facilitates trust and operational validation.

Table 6 presents the performance of the proposed model on majority and minority classes in the NSL-KDD and MAWI datasets. This highlights how off-policy PPO mitigates class imbalance. These results confirm the ability of off-policy PPO to enhance sensitivity to minority class predictions while maintaining strong performance on the majority class. By integrating a reward strategy that emphasizes correct minority classifications, the model ensures fairer outcomes and improved generalizability. This is crucial for real-world anomaly detection in imbalanced metaverse scenarios.

**Table 6.** Performance evaluation of the proposed model on majority and minority classes for the NSL-KDD and MAWI datasets

Metric	NSL-KDD		MAWI	
	Majority	Minority	Majority	Minority
Accuracy	88.348 ± 0.042	85.632 ± 0.063	92.425 ± 0.053	89.326 ± 0.063
F-measure	87.236 ± 0.058	83.426 ± 0.063	89.163 ± 0.038	86.726 ± 0.09
G-means	88.103 ± 0.064	84.303 ± 0.048	89.935 ± 0.081	87.825 ± 0.020
AUC	0.882 ± 0.052	0.846 ± 0.063	0.880 ± 0.012	0.861 ± 0.023

AUC, area under the curve

#### Analysis of the BOHB algorithm

This section evaluates the BOHB algorithm against 9 well-known hyperparameter optimization techniques. For fairness, all model variables were standardized during the testing process. The comparison includes 3 conventional methods: random search, grid search, and BO. It also considers 6 evolutionary algorithms: salp swarm algorithm [64], human mental search [65], bat algorithm [66], firefly algorithm [67], artificial bee colony [68], and differential evolution [68]. In summary, BOHB demonstrates robust and scalable hyperparameter tuning performance across datasets, substantially outperforming traditional and evolutionary approaches. Its effectiveness and efficiency make it highly suitable for complex real-world scenarios, such as the metaverse, requiring high accuracy and resource-aware optimization.

As shown in Table 7, the superiority of the BOHB method over traditional and modern hyperparameter optimization techniques is statistically validated using paired *t* tests on the NSL-KDD and MAWI datasets. Comparisons against random search, grid search, BO, and several evolutionary algorithms show consistently substantial improvements, with *P* values < 0.05 across all key metrics. For the NSL-KDD dataset, BOHB achieved a 2.433% higher accuracy and a 4.5% increase in AUC compared to BO (P=0.001 for both). Similarly, on the MAWI dataset, BOHB outperformed BO by 5.649% in accuracy (P=0.001) and 2.2% in AUC (P=0.002). Confidence intervals further reinforce these findings, with BOHB’s accuracy gains ranging from 2.1% to 2.7% for NSL-KDD and from 5.3% to 6.0% for MAWI.

**Table 7.** Performance metrics comparison of basic and metaheuristic techniques for hyperparameter optimization across the NSL-KDD and MAWI datasets

Model	Accuracy	F-measure	G-means	AUC
NSL-KDD				
Random search	72.603 ± 0.074	72.650 ± 0.035	73.432 ± 0.089	0.708 ± 0.093
Grid search	73.330 ± 0.062	74.223 ± 0.027	74.959 ± 0.049	0.718 ± 0.062
Bayesian optimization	85.572 ± 0.021	86.236 ± 0.062	86.453 ± 0.053	0.825 ± 0.041
Hyperband	84.370 ± 0.028	84.794 ± 0.074	85.456 ± 0.054	0.818 ± 0.087
SSA	74.432 ± 0.057	75.608 ± 0.062	76.263 ± 0.064	0.733 ± 0.072
HMS	75.592 ± 0.041	76.630 ± 0.087	77.227 ± 0.012	0.739 ± 0.086
BA	77.321 ± 0.037	78.571 ± 0.094	78.553 ± 0.019	0.747 ± 0.066
FA	78.750 ± 0.071	79.490 ± 0.035	79.497 ± 0.088	0.763 ± 0.064
ABC	79.801 ± 0.009	81.463 ± 0.079	81.263 ± 0.067	0.771 ± 0.064
DE	80.888 ± 0.059	83.553 ± 0.068	82.246 ± 0.061	0.777 ± 0.041
BOHB	**88.005 ± 0.090**	**87.271 ± 0.033**	**87.986 ± 0.001**	**0.870 ± 0.011**
MAWI				
Random search	73.478 ± 0.034	73.300 ± 0.025	73.907 ± 0.041	0.718 ± 0.088
Grid search	74.823 ± 0.094	74.153 ± 0.081	74.720 ± 0.067	0.725 ± 0.058
Bayesian optimization	86.535 ± 0.012	84.226 ± 0.059	87.334 ± 0.027	0.851 ± 0.021
Hyperband	85.671 ± 0.049	85.115 ± 0.069	85.724 ± 0.032	0.841 ± 0.064
SSA	76.264 ± 0.007	77.153 ± 0.010	75.721 ± 0.031	0.731 ± 0.025
HMS	77.057 ± 0.021	76.267 ± 0.033	76.723 ± 0.018	0.747 ± 0.032
BA	78.160 ± 0.024	78.031 ± 0.020	78.463 ± 0.019	0.763 ± 0.033
FA	79.228 ± 0.057	79.963 ± 0.050	78.807 ± 0.054	0.772 ± 0.033
ABC	82.801 ± 0.023	83.270 ± 0.063	83.013 ± 0.022	0.790 ± 0.073
DE	81.697 ± 0.049	79.956 ± 0.040	81.181 ± 0.048	0.802 ± 0.050
BOHB	**92.184 ± 0.089**	**88.992 ± 0.089**	**89.738 ± 0.084**	**0.873 ± 0.010**

AUC, area under the curve; SSA, salp swarm algorithm; HMS, human mental search; BA, bat algorithm; FA, firefly algorithm; ABC, artificial bee colony; DE, differential evolution; BOHB, Bayesian Optimization Hyperband

Table 8 compares the computational efficiency of BOHB with other hyperparameter optimization methods, focusing on runtime and GPU usage for the NSL-KDD and MAWI datasets. These results highlight BOHB’s ability to deliver fast and efficient hyperparameter tuning. This makes it well-suited for large-scale and resource-constrained environments without compromising optimization quality.

**Table 8.** Performance metrics comparison of various hyperparameter optimization methods across the NSL-KDD and MAWI datasets

Algorithm	NSL-KDD		MAWI	
	Runtime (s)	GPU (GB)	Runtime (s)	GPU (GB)
Random search	2,560 ↑282	18.6 ↑4.3	2,635 ↑268	19.5 ↑4.3
Grid search	3,145 ↓303	24.1 ↓1.2	3,268 ↓365	25.3 ↓1.5
Bayesian opt.	3,056 ↓214	25.9 ↓3.0	3,156 ↓253	26.4 ↓2.6
Hyperband	2,636 ↑206	20.2 ↑2.7	2,706 ↑197	21.3 ↑2.5
SSA	2,936 ↓94	26.2 ↓3.3	3,026 ↓123	27.8 ↓4.0
HMS	3,012 ↓170	25.1 ↓2.2	3,156 ↓253	26.0 ↓2.2
BA	2,963 ↓121	25.4 ↓2.5	3,068 ↓165	26.7 ↓2.9
FA	2,923 ↓81	25.7 ↓2.8	3,003 ↓100	26.1 ↓2.3
ABC	3,176 ↓334	26.2 ↓3.3	3,256 ↓353	26.0 ↓2.2
DE	3,069 ↓227	26.4 ↓3.5	3,108 ↓205	27.9 ↓4.1
**BOHB (ours)**	**2,842**	**22.9**	**2,903**	**23.8**

GPU, graphics processing unit; SSA, salp swarm algorithm; HMS, human mental search; BA, bat algorithm; FA, firefly algorithm; ABC, artificial bee colony; DE, differential evolution; BOHB, Bayesian Optimization Hyperband

**Fig. 13.**
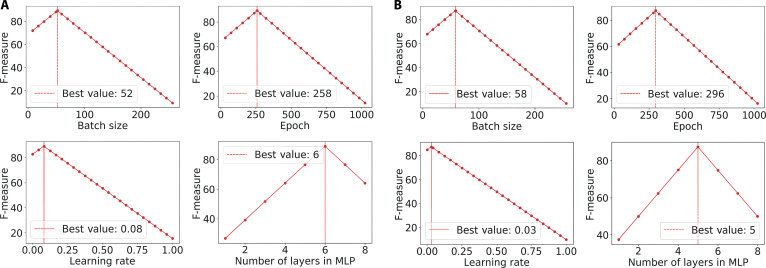
Hyperparameter optimization analysis across the (A) NSL-KDD and (B) MAWI datasets.

Figure 13 demonstrates BOHB’s capability to fine-tune hyperparameters for optimal F-measure performance on the NSL-KDD and MAWI datasets. These adaptive selections optimize learning without overfitting or excessive computation. The consistent improvements validate BOHB’s strength in navigating complex hyperparameter spaces and tailoring configurations to diverse data conditions.

#### Key findings summary

**Fig. 14.**
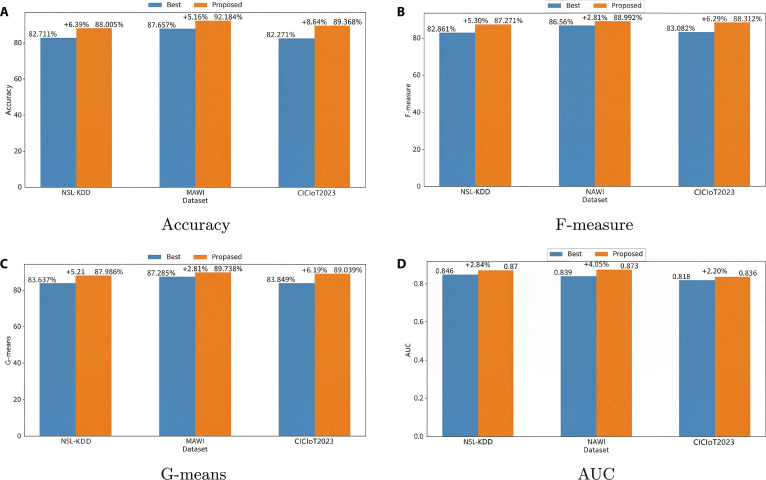
Comparative performance of the proposed model vs. best-existing models on the NSL-KDD, MAWI, and CICIoT2023 dataset: (A) Accuracy, (B) F-measure, (C) G-means, and (D) AUC.

Figure 14 and Table 9 compare the proposed model with existing methods. On the NSL-KDD dataset, it achieved 88.005% accuracy, improving upon IoMT by 6.39%, with F-measure, G-means, and AUC gains of 5.30%, 5.21%, and 2.84%, respectively. On the MAWI dataset, the model achieved 92.184% accuracy—5.16% higher than IoMT—with respective metric improvements of 2.81%, 2.81%, and 4.05%. On the CICIoT2023 dataset, accuracy rose by 8.64%, and F-measure, G-means, and AUC improved by 6.29%, 6.19%, and 2.20%, respectively.

**Table 9.** Comparative performance of the proposed model vs. best-existing models on NSL-KDD, MAWI, and CICIoT2023 datasets

Model	NSL-KDD		MAWI		CICIoT2023	
	Best previous	Proposed model	Best previous	Proposed model	Best previous	Proposed model
Accuracy	IoMT: 82.711%	88.005% ↑6.39%	IoMT: 87.657%	92.184% ↑5.16%	IoMT: 82.271%	89.368% ↑8.64%
F-measure	IoMT: 82.881%	87.271% ↑5.30%	IoMT: 86.560%	88.992% ↑2.81%	IoMT: 83.085%	88.312% ↑6.29%
G-means	IoMT: 83.637%	87.986% ↑5.21%	IoMT: 87.285%	89.738% ↑2.81%	IoMT: 83.849%	89.039% ↑6.19%
AUC	IoMT: 0.846	0.870 ↑2.84%	IoMT: 0.839	0.873 ↑4.05%	IoMT: 0.818	0.836 ↑2.20%

AUC, area under the curve

The superiority of the proposed method can be attributed to the synergy between advanced reinforcement learning, explainable feature selection, and efficient optimization. The approach integrates off-policy PPO with SHAP-based feature selection. It also includes a reward function tailored to prioritize the minority anomaly class. This design addresses both high-dimensional data and class imbalance in metaverse healthcare anomaly detection. PPO enables adaptive policy learning in dynamic environments, which suits the unpredictable nature of real-time 6G-enabled virtual healthcare. SHAP ensures interpretability and relevance in feature selection, critical for high-stakes medical decisions. BOHB was selected for its proven efficiency in hyperparameter optimization across noisy, expensive-to-evaluate objective functions. Compared to conventional methods such as RNN-IoT and IoMT, this hybrid model offers better adaptability, scalability, and performance in imbalanced, multimodal metaverse data streams.

## Conclusion

This paper presents a novel anomaly detection approach that effectively addresses major cybersecurity challenges in metaverse healthcare. This method leverages 6G technology and combines off-policy PPO for feature selection and class imbalance handling with BOHB for hyperparameter optimization. Specifically, the PPO framework is integrated with SHAP-based feature selection to identify the most informative input features. The PPO reward function is adjusted using a class-priority mechanism that increases the learning focus on minority anomaly samples, improving class balance during training.

Our experiments on the NSL-KDD, MAWI, and CICIoT2023 datasets clearly demonstrate that the proposed approach outperforms leading models, achieving F-measures of 87.27%, 88.99%, and 88.31%, respectively. These findings confirm the practicality of the model in detecting threats in immersive metaverse healthcare systems. These systems involve real-time patient data flowing across interconnected virtual environments powered by 6G technology. The proposed PPO-BOHB architecture is well-suited for dynamic healthcare scenarios. These include virtual reality consultations, digital twins, and IoT-based patient monitoring. By adapting to the class imbalance inherent in such heterogeneous data streams, the model enables more secure and responsive healthcare delivery in metaverse settings. This strengthens the argument for domain-specific ML designs in future virtual medical infrastructures.

Despite the strong empirical results, a limitation of this work lies in the datasets used for evaluation. The NSL-KDD, MAWI, and CICIoT2023 datasets are reliable benchmarks for network intrusion and IoT traffic analysis. However, they do not fully capture the communication patterns, privacy constraints, or multimodal data flows typical of real metaverse healthcare environments. Therefore, the findings should be interpreted as a proof of concept rather than direct evidence of clinical applicability. Future validation on healthcare-specific datasets, incorporating physiological, behavioral, and sensor-based signals, is necessary to confirm the relevance of the model in real-world scenarios.

In future work, several avenues are envisioned to further enhance the capabilities and applications of the proposed anomaly detection model. Future efforts should adapt the model for real-time operation, given the dynamic nature of 6G networks and the metaverse. This would require improving the computational efficiency of off-policy PPO and BOHB so they can effectively process live data streams. Real-time anomaly detection is crucial for swift threat mitigation in healthcare settings, where prompt action is essential for ensuring patient safety and data security. Another area for future research is integrating federated learning into the model to address the data privacy concerns prevalent in the healthcare sector. This approach would enable the model to learn from decentralized data sources without transferring sensitive data centrally. This would improve privacy and ensure compliance with strict data protection regulations. It also enables the model to learn from a wider range of data sources, potentially increasing its robustness and accuracy in detecting anomalies across different healthcare environments. Additionally, future research could explore the integration of large language models (LLMs) to enhance the semantic understanding of communication patterns within metaverse healthcare environments. LLMs can support anomaly detection by interpreting unstructured data such as clinical notes or patient-generated inputs, providing a more holistic threat assessment.

## Materials and Methods

### Dataset

To evaluate the proposed model, we use the following dataset:

**NSL-KDD** [69]: An enhanced version of the KDD Cup 1999 dataset, NSL-KDD eliminates duplicate entries and balances class distributions. It includes KDDTrain+, KDDTest+, and KDDTest-21 subsets, covering attack types such as DoS, Probe, remote to local, and user to root [70].

**MAWI** [71]: This dataset comprises anonymized 15-min daily traffic traces from a trans-Pacific link between Japan and the United States. Collected since 2001, it reflects real-world network behavior across evolving conditions.

**CICIoT2023** [39]: To evaluate real-world generalizability, this dataset includes 33 attack types across 105 IoT devices, grouped into 7 categories (e.g., DDoS, spoofing, and Mirai). It contains 169 packet capture and comma-separated values files, each with 46 features. The distribution of attack types is uneven. Web-based and brute-force attacks are underrepresented, highlighting the imbalance and making them crucial for validating anomaly detection in real IoT scenarios.

These datasets were selected because they collectively capture the evolving complexity and heterogeneity of IoT-enabled metaverse healthcare environments. The NSL-KDD dataset provides a balanced foundation for benchmarking intrusion detection performance under controlled conditions, making it suitable for initial calibration of the proposed RL-based model. The MAWI dataset complements this by providing real-world backbone traffic traces. These traces simulate variability, burstiness, and noise patterns commonly seen in healthcare communication networks. Finally, CICIoT2023 offers the most realistic testing ground. It includes diverse IoT traffic patterns, modern attack types, and device-level behavioral data. These reflect the interactions between patients, sensors, and cloud infrastructure in a metaverse-based healthcare system. Together, these datasets enable a layered evaluation approach, progressing from structured experimentation to real-world validation, ensuring that the proposed framework remains both generalizable and robust for anomaly detection in metaverse healthcare ecosystems.

For training, validation, and testing, each dataset is divided using a stratified split strategy, with proportions of 70% for training, 15% for validation, and 15% for testing. Stratification maintains a balanced class distribution across all data subsets. This is crucial for avoiding bias during reinforcement learning and model fine-tuning. The training set is used for parameter optimization and reward adaptation, while the validation set monitors convergence and mitigates overfitting through early stopping. The independent test set evaluates the generalization of the final model under unseen conditions. This split ratio follows prior research in anomaly detection. Keeping at least 15% for validation improves tuning stability, especially when BOHB modifies configuration ranges during training. This design supports reproducibility and consistent benchmarking across datasets of varying size and imbalance levels.

### Evaluation metrics

To evaluate the proposed model, we used accuracy, F-measure, G-means, and AUC metrics. Accuracy provides a general overview of model correctness, but can be misleading in imbalanced datasets. The F-measure combines precision and recall to indicate how effectively the model detects anomalies in the minority class. This is particularly important in IoT-enabled metaverse healthcare. G-means evaluates the balance between sensitivity and specificity, ensuring that both normal and anomalous classes are accurately represented. This is particularly essential in real-time healthcare systems, where false negatives can compromise patient safety. The AUC provides a threshold-independent evaluation of performance. It reflects how well the model separates normal and anomalous behavior under class imbalance. Together, these metrics offer a comprehensive evaluation aligned with the high-stakes demands of healthcare anomaly detection.

The accuracy, F-measure, and G-means metrics are mathematically defined as follows:

$$\text{Accuracy}=\frac{\mathrm{TP}+\mathrm{TN}}{\text{Total}\;\text{observations}}$$ (22)

$$F-\text{measure}=2\times \frac{\text{Precision}\times \text{Recall}}{\text{Precision}+\text{Recall}}$$ (23)

$$G-\text{means}=\sqrt{\text{Recall}\times \text{Specificity}}$$ (24)

where:

$$\text{Precision}=\frac{\mathrm{TP}}{\mathrm{TP}+\mathrm{FP}}$$ (25)

$$\text{Recall}=\frac{\mathrm{TP}}{\mathrm{TP}+\mathrm{FN}}$$ (26)

$$\text{Specificity}=\frac{\mathrm{TN}}{\mathrm{TN}+\mathrm{FP}}$$ (27)

where TP (true positive) refers to the number of correctly identified anomalies, meaning the model successfully detected actual abnormal events. FP (false positive) refers to normal behaviors that are incorrectly classified as anomalies. Such errors may trigger unnecessary alerts or actions. TN (true negative) represents normal events correctly identified by the model, showing that it performs reliably under normal operating conditions. FN (false negative) refers to cases where the model fails to detect actual anomalies.

## Data Availability

The data that support the findings of this study are available from the corresponding authors upon reasonable request.
